# Supplementary material for: Factors influencing early sexual initiation among hill tribe youths in Chiang Rai Province, Northern Thailand: A community-based cross-sectional study
Source: PLoS One. 2025 Apr 8;20(4):e0321083. doi: 10.1371/journal.pone.0321083 (PMC11991288; doi:10.1371/journal.pone.0321083)
Supplement: S1 File — A validated questionnaire consisted of four parts with a total of 73 questions. (PDF) [file pone.0321083.s001.pdf]

NO.________

แบบประเมินพฤติกรรมเสี่ยงต่อการติดเชื้อเอดส์และการพัฒนารูปแบบการป้องกันเอดส์

ในเยาวชนในกลุ่มชนชาติพันธุ์ภาคเหนือของประเทศไทย

น้ำหนัก ....กิโลกรัม
ส่วนสูง ....เซนติเมตร
ความดันโลหิต ....mmHg
ชีพจร.......................................ครั้ง/นาที

ส่วนที่ 1 ข้อมูลทั่วไป

1.1 เพศ

☐ ชาย ☐ หญิง

1.2 อายุ……..... ปี

1.3 ชนเผ่า

☐ อาข่า ☐ ลาหู่ ☐ ม้ง ☐ เย้า ☐ กะเหรี่ยง ☐ ลีซู ☐ อื่นๆ ระบุ…………

1.4 สถานภาพสมรส

☐ โสด ☐ สมรส (........ครั้ง) ☐ หม้าย ☐ หย่า ☐ แยกกันอยู่

1.5 ท่านแต่งงานครั้งแรกตอนอายุ...........ปี

1.6 ศาสนา

☐ พุทธ (ผีบรรพบุรุษ) ☐ คริสต์ ☐ อิสลาม ☐ อื่นๆ (ระบุ)……

1.7 ระดับการศึกษา

☐ ไม่ได้เรียน ☐ ประถมศึกษา ☐ มัธยมศึกษา ☐ ปวช./ปวส./อนุปริญญา ☐ ปริญญาตรี ☐ อื่นๆ ระบุ………..

1.8 อาชีพหลักปัจจุบัน

☐ ว่างงาน ☐ นักเรียน/นักศึกษา ☐ เกษตรกร ☐ รับจ้าง ☐ ค้าขาย/ธุรกิจส่วนตัว ☐ อื่นๆ ระบุ.............

1.9 ท่านมีรายได้หรือไม่

☐ ไม่มี

☐ มี จำนวน…………………บาทต่อเดือน/ปี ☐ ไม่สามารถคำนวณได้

1.10 จำนวนสมาชิกในครอบครัวของท่านที่อยู่ด้วยกันทั้งหมด………………………คน

1.11 ปัจจุบันท่านอาศัยหลักๆอยู่ที่ใด

☐ บ้าน ☐ หอพัก ☐ อาศัยอยู่กับญาติ ☐ อื่นๆ ระบุ…………..

1.12 อาศัยอยู่กับใคร

☐ อยู่คนเดียว ☐ พ่อและแม่ ☐ พ่อ ☐ แม่ ☐ ญาติ ☐พี่/น้อง ☐ เพื่อน ☐ แฟน ☐ นายจ้าง

1.13 สถานภาพสมรสของพ่อแม่

☐ อยู่ด้วยกัน ☐ หย่า ☐ หม้าย ☐ แยกกันอยู่

1.13.1 พ่อและแม่ของท่านทะเลาะกันหรือไม่

☐ ไม่ทะเลาะ ☐ ทะเลาะบางครั้ง ☐ ทะเลาะบ่อย

ส่วนที่ 2 พฤติกรรมสุขภาพ

2.1 ท่านสูบบุหรี่หรือไม่

☐ สูบ..............ปี ☐ เคยสูบ…………ปี ☐ ไม่เคยสูบหรือแค่เคยลองครั้งสองครั้ง

2.2 ท่านดื่มสุราหรือไม่

☐ ดื่ม.............ปี ☐ เคยดื่ม…………ปี ☐ ไม่เคยดื่มหรือแค่เคยลองครั้งสองครั้ง

2.3 ท่านเสพยาบ้าหรือไม่

☐ เสพ.............ปี ☐ เคยเสพ………..ปี ☐ ไม่เคยเสพหรือแค่เคยลองครั้งสองครั้ง

2.4 ท่านฉีดเฮโรอีนหรือไม่

☐ ฉีด.............ปี ☐ เคยฉีด…………..ปี ☐ ไม่เคยฉีดหรือแค่เคยลองครั้งสองครั้ง

2.5 ท่านเสพยาไอซ์หรือไม่

☐ เสพ.............ปี ☐ เคยเสพ………..ปี ☐ ไม่เคยเสพหรือแค่เคยลองครั้งสองครั้ง

2.6 ท่านสูบฝิ่นหรือไม่

☐ สูบ.............ปี ☐ เคยสูบ……….....ปี ☐ ไม่เคยสูบหรือแค่เคยลองครั้งสองครั้ง

2.7 ท่านสูบกัญชาหรือไม่

☐ สูบ.............ปี ☐ เคยสูบ…………..ปี ☐ ไม่เคยสูบหรือแค่เคยลองครั้งสองครั้ง

2.8 ท่านเคยได้รับการบำบัดยาเสพติดหรือไม่

☐ อยู่ในระหว่างการบำบัด ☐ เคยได้รับการบำบัด จำนวน……………ครั้ง ☐ ไม่เคย ☐ ไม่อยากตอบ

2.9 ท่านเคยได้รับวัคซีนป้องกันไวรัสตับอักเสบหรือไม่

☐ มี ☐ ไม่มี ☐ ไม่แน่ใจ ☐ ไม่ทราบ

2.10 สมาชิกในครอบครัวของท่านแค่เป็นโรคไวรัสตับอักเสบหรือไม่

☐ มี ☐ ไม่มี ☐ ไม่แน่ใจ ☐ ไม่ทราบ

ข้อ	คำถาม	เคย	ไม่เคย
2.11	ท่านเคยสักตามร่างกายหรือไม่		
2.12	ท่านเคยการเจาะหูหรือไม่		
2.13	ท่านเคยได้รับการเติมเลือดหรือไม่		
2.14	ท่านเคยได้รับการเปลี่ยนถ่ายอวัยวะหรือไม่		
2.15	ท่านเคยได้รับการผ่าตัดหรือไม่		
2.16	ท่านเคยได้รับการฉีดยาจากหมอเถื่อนหรือไม่		
2.17	ท่านเคยได้รับการฝังเข็มหรือไม่		
2.18	ท่านเคยใช้แปรงสีฟันร่วมกับคนอื่นหรือไม่		

ส่วนที่ 3 พฤติกรรมเสี่ยง

3.1 ท่านเคยทำงานแหล่งต่อไปนี้หรือไม่

3.1.1 นอกหมู่บ้าน ☐ เคย…….….เดือน/ปี ☐ ไม่เคย

3.1.2 ในตัวเมืองอำเภอหรือตัวเมืองเชียงราย ☐ เคย…….….เดือน/ปี ☐ ไม่เคย

3.1.3 ต่างจังหวัด ☐ เคย…..…….เดือน/ปี ☐ ไม่เคย

3.1.4 ต่างประเทศ ☐ เคย…….….เดือน/ปี ☐ ไม่เคย

3.2 ท่านเคยมีเพศสัมพันธ์หรือไม่ (ถ้าไม่มี ข้ามไป ส่วนที่ 4)

☐ มี ☐ ไม่มี ☐ ไม่อยากตอบ

3.3 ท่านมีเพศสัมพันธ์ครั้งแรกตอนอายุ.................ปี

3.4 ท่านมีเพศสัมพันธ์ครั้งแรกกับใคร

☐ แฟน ☐ สามี/ภรรยา ☐ หญิงขายบริการทางเพศ ☐ ชายขายบริการทางเพศ ☐ คนที่เพิ่งรู้จัก

3.5 ท่านมีแฟนไหม

☐ มี ☐ ไม่มี

3.6 ท่านเคยมีเพศสัมพันธ์กับแฟนคนปัจจุบันหรือไม่

☐ มี ☐ ไม่มี ☐ ไม่อยากตอบ

3.7 ในช่วง 1 ปีที่ผ่านมา ถ้าท่านเคยมีเพศสัมพันธ์กับคนที่เพิ่งรู้จัก แต่ไม่ใช่ผู้หญิง/ชายบริการทางเพศ หรือไม่

☐ มี ☐ ไม่มี ☐ ไม่อยากตอบ

3.8 ในช่วง 1 ปี ที่ผ่านมาท่านเคยมีเพศสัมพันธ์กับหญิง/ชายขายบริการทางเพศหรือไม่

☐ มี ☐ ไม่มี ☐ ไม่อยากตอบ

3.9 ท่านดื่มสุราหรือสารเสพติดก่อนมีเพศสัมพันธ์ไหม

☐ ไม่มี ☐ มี

ส่วนที่ 6 ความรู้ ทัศนคติ

ความรู้

ข้อคำถาม	ถูก	ผิด	ไม่แน่ใจ
1. การใช้กรรไกรตัดเล็บร่วมกันทำให้ติดเชื้อเอดส์ได้			
2. โรคเอดส์ติดต่อกันจากการจูบกัน			
3. การมีเพศสัมพันธ์กับผู้ติดเชื้อ แม้จะใช้ถุงยางอนามัยก็ติดโรคเอดส์ได้			
4. โรคเอดส์ สามารถติดได้จากการโดนยุงกัด			
5. เราสามารถติดเอดส์จากการใช้เข็มฉีดยาร่วมกันกับบุคคลที่มีเชื้อเอดส์			
6. เราสามารถติดเชื้อเอดส์ จากการรับประทานอาหารร่วมกันกับบุคคลที่มีเชื้อเอดส์ได้			
7. ลูกในครรภ์สามารถติดเอดส์จากแม่ที่ติดเชื้อเอดส์ได้			
8. ผู้หญิงที่ติดเชื้อเอดส์สามารถติดไปสู่ลูกผ่านทางการดื่มนมแม่ได้			
9. โรคเอดส์สามารถรักษาให้หายขาดได้			
10. การติดเชื้อเอดส์ทำให้ภูมิต้านทานของร่างกายต่ำลงและเกิดโรคแทรกซ้อนได้ง่าย			

ทัศนคติ

ประเด็น	เห็นด้วย	ไม่แน่ใจ	ไม่เห็นด้วย
1. การกินข้าวร่วมกับกับผู้ติดเชื้อเอดส์ไม่เป็นไร			
2. เราไม่ควรใช้ห้องน้ำร่วมกับผู้ติดเชื้อเอดส์			
3. ผู้ป่วยโรคเอดส์สามารถทำงานร่วมกับผู้อื่นได้			
4. เราสามารถดูแลผู้ที่ติดโรคเอดส์ในบ้านได้			
5. เราควรเก็บเรื่องการติดเชื้อเอดส์ของบุคคลในครอบครัวให้เป็นความลับ			
6. โรคเอดส์เป็นโรคที่น่ารังเกียจ			
7. เราควรตรวจหาเชื้อเอดส์ก่อนแต่งงาน			
8. คนที่ติดเชื้อเอดส์ทุกคนเป็นคนสำส่อน			

9. เมื่อตรวจพบเชื้อเอดส์ ต้องซื้อยามากินเองก็ได้			
10. เราไม่ควรคบค้าสมาคมกับผู้ติดเอดส์			

NO.________

An assessment of risk behaviors to HIV/AIDS among hill tribe youths in northern Thailand

Weight ....kg
Height ....cm
Blood pressure ....mmHg
Pulse..ครั้ง/นาที

Part 1 General information

1.1 Gender

☐ Male ☐ Female

1.2 Age……... years

1.3 Tribe

☐ Akha ☐ Lahu ☐ Hmong ☐ Yao ☐ Karen ☐ Lisu

1.4 Marital status

☐ Single ☐ Married ☐ Divorced ☐ Widow ☐ Separate

1.5 Age of first marriage............ years

1.6 Religion

☐ Buddhism ☐ Christian ☐ Islam

1.7 Education

☐ Illiterate ☐ Primary school ☐ High school ☐ Vocational school ☐ University

1.8 Occupation

☐ Unemployed ☐ Student ☐ Farmer ☐ Labor ☐ Merchant

1.9 Income

☐ Yes ☐ No

1.10 Number of family member ………………………persons

1.11 Where do you live currently?

☐ House ☐ Dormitory ☐ Stay with cousin

1.12 Who do you live with?

☐ Alone ☐ Father and Mother ☐ Father ☐ Mother ☐ Cousin ☐Brother/Sister

☐ Friends ☐ Boy/Girlfriend ☐ Employer

1.13 Marital status of parents

☐ Married ☐ Divorced ☐ Widow ☐ Separate

1.13.1 Your parents fighting?

☐ No ☐ Sometime ☐ Often

Part 2 Health behaviors

2.1 Smoking

☐ Yes...............years ☐ Ever…………years ☐ No

2.2 Alcohol use

☐ Yes...............years ☐ Ever…………years ☐ No

2.3 Amphetamine use

☐ Yes..............years ☐ Ever………..years ☐ No

2.4 Heroin use

☐ Yes..............years ☐ Ever………..years ☐ No

2.5 Crystal methamphetamine use

☐ Yes..............years ☐ Ever………..years ☐ No

2.6 Opium use

☐ Yes..............years ☐ Ever………..years ☐ No

2.7 Marijuana

☐ Yes..............years ☐ Ever………..years ☐ No

2.8 Have you ever treated from drug use?

☐ On treatment ☐ Ever ☐ No ☐ No answer

2.9 Hepatitis B Vaccine

☐ Yes ☐ No ☐ Not sure ☐ Don't know

2.10 A family member is living with or has lived with hepatitis B

☐ Yes ☐ No ☐ Not sure ☐ Don't know

No.	Questions	Yes	No
2.11	Tattoo		
2.12	Pierced ears		
2.13	Blood-filled		
2.14	Organ transplantation		
2.15	Surgery		
2.16	Being injected by quack		
2.17	Acupuncture		
2.18	Share toothbrushes		

Part 3 Risk behaviors

3.1 Have you ever been working in a……...?

3.1.1 Outside village ☐ Yes ……..….month/year ☐ No

3.2.2 In the city ☐ Yes ……..…. month/year ☐ No

3.3.3 In other provinces ☐ Yes …..……. month/year ☐ No

3.4.4 Experience abroad ☐ Yes ……..…. month/year ☐ No

3.2 Have you ever had sexual intercourse? (If "No" move to part.4)

☐ Yes ☐ No ☐ No answer

3.3 When did you have first sexual intercourse? At age of.................years old.

3.4 Do you have a boyfriend or girlfriend?

☐ Yes ☐ No

3.4 Did you have had sexual intercourse with your boyfriend or girlfriend?

☐ Yes ☐ No

3.5 Did you have sexual experience with one nightstand?

☐ Yes ☐ No

3.6 Did you have sexual experience with prostitute one year prior?

☐ Yes ☐ No

3.7 Do you have a regular partner?

☐ Yes ☐ No

3.8 Did you use alcohol or drug use prior having sexual intercourse?

☐ Yes ☐ No

Part 4 Knowledge and Attitude

Knowledge

Question	True	False	Not sure
1. Using nail clippers together can lead to HIV infection.			
2. Can people transmit HIV through kissing			
3. Having sex with an infected person Even using a condom can become infected with AIDS.			
4. HIV/AIDS can be transmitted by mosquito bites.			
5. Can get HIV from sharing syringes with people who have HIV infected.			
6. Can be infected with HIV. From eating together with people with HIV infected.			
7. Can HIV be passed to an unborn baby in pregnancy			
8. Women infected with HIV can infect their children through breast milk.			
9. Is there a cure for HIV/AIDS?			
11. HIV/AIDS infection lowers the body's immune system and causes complications.			

Attitude

Question	Agree	Not sure	Disagree
1. Eating with people living with AIDS is fine.			
2. We should not use the bathroom with someone infected with AIDS.			
3. HIV infected people can work with others.			
4. Can take care of people with AIDS in the home.			
5. Should keep the HIV infection of family member's secret.			
6. Aids is a stigma disease			
7. Should test for HIV before marriage.			
8. All people had AIDs infected are promiscuous.			
9. If you've been diagnosed with HIV, you can find medicine to eat by yourself.			

10. Should not relationship with people with AIDS.			
