# Supplementary material for: Factors influencing early sexual initiation among hill tribe youths in Chiang Rai Province, Northern Thailand: A community-based cross-sectional study
Source: PLoS One. 2025 Apr 8;20(4):e0321083. doi: 10.1371/journal.pone.0321083 (PMC11991288; doi:10.1371/journal.pone.0321083)
Supplement: Attachment — Submitted filename: Point-by-point response.pdf [file pone.0321083.s005.pdf]

Repose to reviewers and editor comments

Dear Editor of Ploseone,

Thank you very much or the opportunity to improve our paper with excellent comments from reviewer. We have carefully revised by re-analysis in the whole process based in the conditions suggested by reviewers.

TK

PONE-D-23-33898

Influencing factors early sexual initiation among hill tribe youths in northern Thailand

PLOS ONE

Dear Editor,

Thank you very much for opportunity to review the manuscript titled " Influencing factors early sexual initiation among hill tribe youths in northern Thailand" for PLOS ONE.

I think that some aspects of this manuscript are interesting. I believe that some major changes are required. Below are my comments about this manuscript, which I hope are some assistances to the authors.

1.The title, author should be specific "in Chiang Rai province"? not representative in northern of Thailand, and may considering add approach ": community based cross sectional approach"

: Thank you, it's improved

2.An abstract

- Author conclusions "A large proportion of the hill tribe youths have experienced early sexual initiation before the age of 15 years. A specific training program should be integrated into the high school study curriculum to protect early sexual initiation behaviors and safe sex, particularly in males. The curriculum should focus on the impact of alcohol and working in unskilled work sectors and potentially early sexual initiation. As the result based author may conclude in community or family based intervention, attitude come from socialization in family….etc. Thailand has a sexual education curriculum at all level in school.

: Thank you for the comment. We know that Thailand has a curriculum at all school levels, but we intend to add an extra and special curriculum. You said that it exists at all levels, but according to the findings of our program, those people still lack skills and knowledge in safe sex. This is the reason that we put this statement together. We apologize if this makes you confused.

: We also agree with you to add family and community levels, which we have added information in the section.

2. Methodology,

- page 5 line 25-27 author mention that “In the section on knowledge of safe sex (10 items), there were classified into three categories; poor (0-5 scored), moderate (6-8 scored), and good (9-10 scored).” It is knowledge about HIV transmission (not safe sex). Please clearly how scoring and range of score and cutting point? Including reference?

: This category is formed by the findings from our previous study in Ref. No.23 and also on our pilot phase. It’s improved.

-Substance use question between Thai and English inconsistent. The answer means? Current use? Ever but quit? Never or ever tried? Please clearly explain at variable section. What yes?

: Thank you for the comment. Based on our developed initial questionnaire, three answers to these questions were provided. However, after piloting, two options were found: better validity and reliability. Then, we used two options to answer these questions. We have revised the questionnaire. Thank you so much for the great comment, which is excellent for those who will apply our original questionnaire in the future. Our update/revised questionnaires (ENG and THAI) are attached.

3. Results

- Table 3 Something error? Author present variable” Alcohol or drug use prior having sexual initiation with 3 answers <no, sometime, yes> but in questionnaire part 3 item 3.9 in English and Thai language the answer only <Yes, No>

: Please see our response in your previous comment.

- Table 1 and 2 author should present as below

characteristics	Total		Early sexual initiation				Never had sexual experience		χ 2	p-value
			Yes (<14)		No (≥15)					
	n	%	n	%	n	%	n	%		

: Thank you so much for your great comment, we agreed with you and have carefully revised tables, please see our new tables 1, 2, and 4

-Table 4 Author definition “Early sexual initiation” yes= age at first sexual initiation <14 and No= age at first sexual initiation ≥15 including never had sexual experience. Author should exclude never had sexual experience or use as reference.

: We thank you so much for such great suggestion!. We have re-run the model by comparing those who had sexual before and after 15 years by excluding those who did not have sexual experience from the model. Please see detail in table 4. The new model really reflects the objective of the study.

4. Discussion

Page 15-line 16-18 Author discuss that “The hill tribe youths have a younger age of their first sexual initiation than Thai youths which might be the culture of the hill tribe that needs to get married early to have a child for help in their family work.” Is it culture issue? Economic or occupational Issue? Author should explain how culture difference from hill tribe and general youths.

: Thank you for your feedback. It’s added the culture issue on page 19-line 9-20.

Thank you so much,

TK

Associate Prof. Dr. Tawatchai Apidechkul, MSc (Infectious Epidemiology), Dr.P.H. (Epidemiology)

School of Health Science, Mae Fah Luang Univeristy, Thailand

Director, Center of Excellence of Hill Tribe Health Research

Former Hubert H Humphrey Fellow (2013-2014), Emory University

Candidate MMSc-GHD, Harvard Medical School, Harvard University, United States
