# Supplementary material for: Factors influencing early sexual initiation among hill tribe youths in Chiang Rai Province, Northern Thailand: A community-based cross-sectional study
Source: PLoS One. 2025 Apr 8;20(4):e0321083. doi: 10.1371/journal.pone.0321083 (PMC11991288; doi:10.1371/journal.pone.0321083)
Supplement: Attachment — Submitted filename: Response to reviewer comment_PAM paper.pdf [file pone.0321083.s006.pdf]

Response to the reviewer comment

PONE-D-23-33898R1
Influencing factors early sexual initiation among hill tribe youths in Chiang Rai Province, northern Thailand: A community-based cross-sectional study
PLOS ONE

Dear Editor and Reviewers,

Thank you very much for the opportunity to revise that paper. We have carefully and completely revised your comments. The paper also double-checked the grammatical error before re-submitting. Please kindly the following our responses.

Thank you,

TK

+++

Please use the space provided to explain your answers to the questions above. You may also include additional comments for the author, including concerns about dual publication, research ethics, or publication ethics. (Please upload your review as an attachment if it exceeds 20,000 characters)

Reviewer #2: Reviewers' comments
Title: Influencing factors early sexual initiation among hill tribe youths in Chiang Rai Province, northern Thailand: A community-based cross-sectional study
The manuscript was well-prepared. I have a few comments as follows.
Abstract
-The conclusion did not come from the study findings. The authors can be more specific, like suggesting what to be include in the training program (what knowledge is lacking from the results).
Methods

: It is a sexual education focused on safe sex and early sexual interaction protection.

-The authors mentioned that the questionnaires were piloted in Mae Chan district. Where did the study take place? Please add in the study setting.

: Thank you. It's added which are Huan Hin Fon and Sun Ti Suk Villages

-Please clarify about the recruitment which should happen without coercion. The authors stated that they contacted village headmen for their permission to access the villages, but how those youth was reached? As most of them were studying, were they

in the village or lived somewhere outside the village for their schooling? Could any youth deny participation? Did their parents, family members, and other villagers know about their entering the study? How many of them refused to join? Any compensation to youth, their family, or villager headmen?

: There was nobody to refuse the participants. In the process of recruitment, after providing them with information about the study, all participants were informed that they had the right to refuse or deny joining the project as the standard protocol for human research ethical procedure. All parents were also informed about the study, and only those who had children aged less than 17 and below were asked for their permission to gather the information from the participants (youths). All participants and parents agreed and were willing to join the project. During the study, we did not have any inclusion and exclusion criteria for whether the participants were in or out of the school. All participants were compensated 200 baht ($6), which is clearly declared in the ethical consideration process.

-Please describe how youth were instructed before completing the questionnaires. Were there any specific guide written in the questionnaire or mentioned verbally, like the time frame for alcohol, smoking, and substance use (lifetime, or any specific duration to be reported), definition of sexual intercourse, etc.)

: Each question provided complete and clear information. We did not have any specific guide for completing the questionnaire; however, all participants were told that if there was any confusion, they could contact the researcher. Nobody asked to clear any questions.

: Since we worked a cross-sectional, then the questions were asked directly with the time frame, except for some question that we had a clear its frame in the questions.

Results

Table 1: The column “Early sexual initiation” can list only “yes” (and add the denominator somewhere). Similarly for variables with yes/no response, presentation in the table can be only yes which would make them easier to read and reduce the table size.

: Thank you for the comments; however, after discussing with the team, we decided to maintain this column and information to make it easier and quicker for the readers to go through the details.

Discussion

Page 18 line 17-18. The study found that a larger proportion of male had early sex initiation than female youth. Was it a result of reporting bias? Males perceived power

over females when they initiated sex, while females might have early sex without their willingness. Thus, males were more likely to report having early sex. It might be related to patriarchy in Thai society, which could be prominent in hill tribe cultures. Just a thought, I know the authors are experts on this issue. Could the authors provide any comments or discussion on this possibility.

: Thank you for this great idea. We had discussed this point while making the conclusion; however, with the study design, we could not extend our thoughts outside of what we found in the results. One of our team is trained in medical anthropology and is trying to work on her project to understand some critical issues from our findings, particularly gender roles and sexual inequality in some tribes. We very much hope that we will be able to publish our findings soon.

Page 19 line 32" the term "poor education" might not appropriate and too broad, given that most participants had attended school. The authors could consider changing to other words like "limited literacy", or "low reading ability".

: Thank you very much for the suggestion; we agree. It's changed it to limited literacy.

Thank you very much,

TK

Assoc Prof. Dr. Tawatchai Apidechkul, MSc (Infectious Epidemiology), Dr. P. H (Epidemiology)

Director, Center of Excellence of Hill Tribe Health Research,

School of Health Science, Mae Fah Luang University, Thailand

Former Hubert H Humphrey Fellow (2013-2014), Emory University
