# Supplementary material for: Factors influencing early sexual initiation among hill tribe youths in Chiang Rai Province, Northern Thailand: A community-based cross-sectional study
Source: PLoS One. 2025 Apr 8;20(4):e0321083. doi: 10.1371/journal.pone.0321083 (PMC11991288; doi:10.1371/journal.pone.0321083)
Supplement: Attachment — Submitted filename: PONE-D-23-33898_R2_AFreviewer.pdf [file pone.0321083.s007.pdf]

PLOS ONE

Influencing factors early sexual initiation among hill tribe youths in Chiang Rai Province, northern Thailand: A community-based cross-sectional study

--Manuscript Draft--

**Manuscript Number:**	PONE-D-23-33898R2
**Article Type:**	Research Article
**Full Title:**	Influencing factors early sexual initiation among hill tribe youths in Chiang Rai Province, northern Thailand: A community-based cross-sectional study
**Short Title:**	Influencing factors early sexual initiation among hill tribe youths in northern Thailand
**Corresponding Author:**	Tawatchai Apidechkul, Dr.P.H.(Epidemiology)<br>Mae Fah Luang University<br>Chiang Rai, THAILAND
**Keywords:**	Early sexual initiation; influencing factors; youth; Hill tribe
**Abstract:**	Background: Early sexual initiation, before the age of 15 years, prior to full physical and mental maturity, could lead to several problems, including sexually transmitted diseases. The study aimed to estimate the prevalence of and determine the factors associated with early sexual initiation among hill tribe youths in Thailand.<br>Methods: A cross-sectional study was applied to gather information using a validated questionnaire from the hill tribe youths aged 15-24 years living in 30 selected hill tribe villages in Chiang Rai Province, Thailand. Data were obtained by self-report in a private and confidential room. Chi-square tests and logistic regression were used to detect the associations between variables at a significance level of a=0.05.<br>Results: A total of 1,310 hill tribe youths participated in the study: 60.8% were females, 59.0% were aged 15-17 years, 34.0% were ~~Akha~~, and 90.1% had no income. Substance use: 22.2% used alcohol, 14.3% smoked, and 2.6% used methamphetamine. A large proportion (79.8%) had poor knowledge of safe sex, and 33.5% had poor attitudes towards early sexual behaviors and safe sex. One-third (30.4%) of the parti[illegible]nts had sexual experience: 37.2% of males and 26.0% of females. One-fourth [illegible]0%) had sexual experience. Among those who had sexual experience, 9.8% had their first sexual initiation before 15 years. The overall age at first sexual initiation was 16.7 years. The minimum age of having first sexual initiation for males was 12 years, and 13 years for females. In the multivariate model, sex and age were found to be associated with early sexual initiation. Males were 7.30 times (95%CI=2.06-25.83) more likely to have experienced early sexual initiation than ~~males~~. Those aged 15-17 were 8.31 times (95%CI=1.68-41.07) more likely to have experienced early sexual initiation than those who were 21-24 years, respectively.<br>Conclusions: A large proportion of the hill tribe youths have sexual experience, and one-tenth have had early sexual initiation before the age of 15 years. Specific knowledge on sexual education programs should be integrated into the high school study curriculum to protect early sexual initiation behaviors and safe sex, particularly in females. Family and community-based interventions should also be strongly recommended for the sexual education training implementation. The curriculum should focus on the impact of substance use and potentially early sexual initiation.
**Order of Authors:**	Ratipark Tamornpark
	Tawatchai Apidechkul, Dr.P.H.(Epidemiology)
	Panupong Upala
	Chalitar Chomchoei
	Fartima Yeemard
**Response to Reviewers:**	Response to the reviewer comment<br>PONE-D-23-33898R1<br>Influencing factors early sexual initiation among hill tribe youths in Chiang Rai Province, northern Thailand: A community-based cross-sectional study<br>PLOS ONE<br>Dear Editor and Reviewers,

Powered by Editorial Manager® and ProduXion Manager® from Aries Systems Corporation

Thank you very much for the opportunity to revise that paper. We have carefully and completely revised your comments. The paper also double-checked the grammatical error before re-submitting. Please kindly the following our responses.
Thank you,
TK
+++
Please use the space provided to explain your answers to the questions above. You may also include additional comments for the author, including concerns about dual publication, research ethics, or publication ethics. (Please upload your review as an attachment if it exceeds 20,000 characters)
Reviewer #2: Reviewers' comments
Title: Influencing factors early sexual initiation among hill tribe youths in Chiang Rai Province, northern Thailand: A community-based cross-sectional study
The manuscript was well-prepared. I have a few comments as follows.
Abstract
-The conclusion did not come from the study findings. The authors can be more specific, like suggesting what to be include in the training program (what knowledge is lacking from the results).
Methods
: It is a sexual education focused on safe sex and early sexual interaction protection.

-The authors mentioned that the questionnaires were piloted in Mae Chan district. Where did the study take place? Please add in the study setting.
: Thank you. It's added which are Huan Hin Fon and Sun Ti Suk Villages

-Please clarify about the recruitment which should happen without coercion. The authors stated that they contacted village headmen for their permission to access the villages, but how those youth was reached? As most of them were studying, were they in the village or lived somewhere outside the village for their schooling? Could any youth deny participation? Did their parents, family members, and other villagers know about their entering the study? How many of them refused to join? Any compensation to youth, their family, or villager headmen?
: There was nobody to refuse the participants. In the process of recruitment, after providing them with information about the study, all participants were informed that they had the right to refuse or deny joining the project as the standard protocol for human research ethical procedure. All parents were also informed about the study, and only those who had children aged less than 17 and below were asked for their permission to gather the information from the participants (youths). All participants and parents agreed and were willing to join the project. During the study, we did not have any inclusion and exclusion criteria for whether the participants were in or out of the school. All participants were compensated 200 baht ($6), which is clearly declared in the ethical consideration process.

-Please describe how youth were instructed before completing the questionnaires. Were there any specific guide written in the questionnaire or mentioned verbally, like the time frame for alcohol, smoking, and substance use (lifetime, or any specific duration to be reported), definition of sexual intercourse, etc.)
: Each question provided complete and clear information. We did not have any specific guide for completing the questionnaire; however, all participants were told that if there was any confusion, they could contact the researcher. Nobody asked to clear any questions.
: Since we worked a cross-sectional, then the questions were asked directly with the time frame, except for some question that we had a clear its frame in the questions.

Results
Table 1: The column "Early sexual initiation" can list only "yes" (and add the denominator somewhere). Similarly for variables with yes/no response, presentation in the table can be only yes which would make them easier to read and reduce the table size.

: Thank you for the comments; however, after discussing with the team, we decided to maintain this column and information to make it easier and quicker for the readers to go through the details.

Powered by Editorial Manager® and ProduXion Manager® from Aries Systems Corporation

	Discussion<br>Page 18 line 17-18. The study found that a larger proportion of male had early sex initiation than female youth. Was it a result of reporting bias? Males perceived power over females when they initiated sex, while females might have early sex without their willingness. Thus, males were more likely to report having early sex. It might be related to patriarchy in Thai society, which could be prominent in hill tribe cultures. Just a thought, I know the authors are experts on this issue. Could the authors provide any comments or discussion on this possibility.<br>: Thank you for this great idea. We had discussed this point while making the conclusion; however, with the study design, we could not extend our thoughts outside of what we found in the results. One of our team is trained in medical anthropology and is trying to work on her project to understand some critical issues from our findings, particularly gender roles and sexual inequality in some tribes. We very much hope that we will be able to publish our findings soon.<br><br>Page 19 line 32" the term "poor education" might not appropriate and too broad, given that most participants had attended school. The authors could consider changing to other words like "limited literacy", or "low reading ability".<br>: Thank you very much for the suggestion; we agree. It's changed it to limited literacy.<br><br>Thank you very much,<br><br>TK<br>Assoc Prof. Dr. Tawatchai Apidechkul, MSc (Infectious Epidemiology), Dr. P. H (Epidemiology)<br>Director, Center of Excellence of Hill Tribe Health Research,<br>School of Health Science, Mae Fah Luang University, Thailand<br>Former Hubert H Humphrey Fellow (2013-2014), Emory University
**Additional Information:**	
**Question**	**Response**
**Financial Disclosure**<br><br>Enter a financial disclosure statement that describes the sources of funding for the work included in this submission. Review the submission guidelines for detailed requirements. View published research articles from *PLOS ONE* for specific examples.<br><br>This statement is required for submission and **will appear in the published article** if the submission is accepted. Please make sure it is accurate.	Yes

Powered by Editorial Manager® and ProduXion Manager® from Aries Systems Corporation

**Funded studies**<br>Enter a statement with the following details:<br>• Initials of the authors who received each award<br>• Grant numbers awarded to each author<br>• The full name of each funder<br>• URL of each funder website<br>• Did the sponsors or funders play any role in the study design, data collection and analysis, decision to publish, or preparation of the manuscript?<br><br>Did you receive funding for this work?	
Please add funding details.<br>as follow-up to "**Financial Disclosure**<br><br>Enter a financial disclosure statement that describes the sources of funding for the work included in this submission. Review the submission guidelines for detailed requirements. View published research articles from *PLOS ONE* for specific examples.<br><br>This statement is required for submission and **will appear in the published article** if the submission is accepted. Please make sure it is accurate.<br><br>**Funded studies**<br>Enter a statement with the following details:<br>• Initials of the authors who received each award<br>• Grant numbers awarded to each author<br>• The full name of each funder<br>• URL of each funder website<br>• Did the sponsors or funders play any role in the study design, data collection and analysis, decision to publish, or preparation of the manuscript?<br><br>Did you receive funding for this work?"	The study was funded by The National Research Council of Thailand and Mae Fah Laung University, Thailand (Grant No.06/2560).
Please select the country of your main research funder (please select carefully as in some cases this is used in fee calculation).	THAILAND - TH

as follow-up to "**Financial Disclosure**<br><br>Enter a financial disclosure statement that describes the sources of funding for the work included in this submission. Review the submission guidelines for detailed requirements. View published research articles from *PLOS ONE* for specific examples.<br><br>This statement is required for submission and **will appear in the published article** if the submission is accepted. Please make sure it is accurate.<br><br>**Funded studies**<br>Enter a statement with the following details:<br>• Initials of the authors who received each award<br>• Grant numbers awarded to each author<br>• The full name of each funder<br>• URL of each funder website<br>• Did the sponsors or funders play any role in the study design, data collection and analysis, decision to publish, or preparation of the manuscript?<br><br>Did you receive funding for this work?"	
**Competing Interests**<br><br>Use the instructions below to enter a competing interest statement for this submission. On behalf of all authors, disclose any competing interests that could be perceived to bias this work—acknowledging all financial support and any other relevant financial or non-financial competing interests.<br><br>This statement is required for submission and **will appear in the published article** if the submission is accepted. Please make sure it is accurate and that any funding sources listed in your Funding Information later in the submission form are also declared in your Financial Disclosure statement.	The authors have declared that no competing interests exist.

View published research articles from *PLOS ONE* for specific examples.<br><br>**NO authors have competing interests**<br><br>Enter: *The authors have declared that no competing interests exist.*<br><br>**Authors with competing interests**<br><br>Enter competing interest details beginning with this statement:<br><br>*I have read the journal's policy and the authors of this manuscript have the following competing interests: [insert competing interests here]*<br><br>* typeset	
**Ethics Statement**<br><br>Enter an ethics statement for this submission. This statement is required if the study involved:<br><br>• Human participants<br>• Human specimens or tissue<br>• Vertebrate animals or cephalopods<br>• Vertebrate embryos or tissues<br>• Field research<br><br>Write "N/A" if the submission does not require an ethics statement.<br><br>General guidance is provided below. Consult the submission guidelines for detailed instructions. **Make sure that all information entered here is included in the Methods section of the manuscript.**	The research proposal, including the tools and procedures of data collection, was approved by the Mae Fah Luang University Research Ethics Committee on Human Research (REH-60030). All participants provided written informed consent. Participants younger than 18 years old were required to ask their parents to sign an informed consent form before data collection began. All processes of data collection were executed in a private and confidential room and performed in accordance with the relevant guidelines and regulations (Declaration of Helsinki).

Powered by Editorial Manager® and ProduXion Manager® from Aries Systems Corporation

**Format for specific study types**<br><br>**Human Subject Research (involving human participants and/or tissue)**<br>• Give the name of the institutional review board or ethics committee that approved the study<br>• Include the approval number and/or a statement indicating approval of this research<br>• Indicate the form of consent obtained (written/oral) or the reason that consent was not obtained (e.g. the data were analyzed anonymously)<br><br>**Animal Research (involving vertebrate animals, embryos or tissues)**<br>• Provide the name of the Institutional Animal Care and Use Committee (IACUC) or other relevant ethics board that reviewed the study protocol, and indicate whether they approved this research or granted a formal waiver of ethical approval<br>• Include an approval number if one was obtained<br>• If the study involved *non-human primates*, add *additional details* about animal welfare and steps taken to ameliorate suffering<br>• If anesthesia, euthanasia, or any kind of animal sacrifice is part of the study, include briefly which substances and/or methods were applied<br><br>**Field Research**<br><br>Include the following details if this study involves the collection of plant, animal, or other materials from a natural setting:<br>• Field permit number<br>• Name of the institution or relevant body that granted permission	
**Data Availability**<br><br>Authors are required to make all data underlying the findings described fully available, without restriction, and from the time of publication. PLOS allows rare exceptions to address legal and ethical concerns. See the PLOS Data Policy and FAQ for detailed information.	Yes - all data are fully available without restriction

A Data Availability Statement describing where the data can be found is required at submission. Your answers to this question constitute the Data Availability Statement and **will be published in the article**, if accepted.<br><br>**Important:** Stating 'data available on request from the author' is not sufficient. If your data are only available upon request, select 'No' for the first question and explain your exceptional situation in the text box.<br><br>Do the authors confirm that all data underlying the findings described in their manuscript are fully available without restriction?	
**Describe where the data may be found in full sentences. If you are copying our sample text, replace any instances of XXX with the appropriate details.**<br><br>• If the data are **held or will be held in a public repository**, include URLs, accession numbers or DOIs. If this information will only be available after acceptance, indicate this by ticking the box below. For example: *All XXX files are available from the XXX database (accession number(s) XXX, XXX.*).<br>• If the data are all contained **within the manuscript and/or Supporting Information files**, enter the following: *All relevant data are within the manuscript and its Supporting Information files.*<br>• If neither of these applies but you are able to provide **details of access elsewhere**, with or without limitations, please do so. For example:<br><br>*Data cannot be shared publicly because of [XXX]. Data are available from the XXX Institutional Data Access / Ethics Committee (contact via XXX) for researchers who meet the criteria for access to confidential data.*<br><br>*The data underlying the results presented in the study are available from (include the name of the third party*	The data underlying the results presented in the study are available from the supplement file attachment (S2).

Powered by Editorial Manager® and ProduXion Manager® from Aries Systems Corporation

and contact information or URL).<br>• This text is appropriate if the data are owned by a third party and authors do not have permission to share the data.<br><br>* typeset	
Additional data availability information:	

Powered by Editorial Manager® and ProduXion Manager® from Aries Systems Corporation

Manuscript

Click here to access/download;Manuscript;Sexual initiation-TKFinaL_Clenaed .docx

Influencing factors early sexual initiation among hill tribe youths in Chiang Rai Province, northern Thailand: A community-based cross-sectional study

Ratipark Tamornpark[1,2], Tawatchai Apidechkul[1, 2, *], Panupong Upala[1,2], Chalitar Chomchoei[1,2], Fartima Yeemard[1]

[1]Center of Excellence for The Hill tribe Health Research, Mae Fah Luang University, Chiang Rai, Thailand

[2]School of Health Science, Mae Fah Luang University, Chiang Rai, Thailand

Authors' email

Ratipark Tamornpark email: ratipark.tam@mfu.ac.th

Tawatchai Apidechkul email: Tawatchai.api@mfu.ac.th

Panupong Upala email: panupong.upa@mfu.ac.th

Chalitar Chomchoei email: chalitar.cho@mfu.ac.th

Fartima Yeemard email: fartima.far052@gmail.com

[*]Correspondence: Tawatchai.api@mfu.ac.th

Abstract

Background: Early sexual initiation, before the age of 15 years, prior to full physical and mental maturity, could lead to several problems, including sexually transmitted diseases. The study aimed to estimate the prevalence of and determine the factors associated with early sexual initiation among hill tribe youths in Thailand.

Methods: A cross-sectional study was applied to gather information using a validated questionnaire from the hill tribe youths aged 15-24 years living in 30 selected hill tribe villages in Chiang Rai Province, Thailand. Data were obtained by self-report in a private and confidential room. Chi-square tests and logistic regression were used to detect the associations between variables at a significance level of α=0.05.

Results: A total of 1,310 hill tribe youths participated in the study: 60.8% were females, 59.0% were aged 15-17 years, 34.0% were Akha, and 90.1% had no income. Substance use: 22.2% used alcohol, 14.3% smoked, and 2.6% used methamphetamine. A large proportion (79.8%) had poor knowledge of safe sex, and 33.5% had poor attitudes towards early sexual behaviors and safe sex. One-third (30.4%) of the participants had sexual experience: 37.2% of males and 26.0% of females. One-fourth (24.0%) had sexual experience. Among those who had sexual experience, 9.8% had their first sexual initiation before 15 years. The overall age at first sexual

initiation was 16.7 years. The minimum age of having first sexual initiation for males was 12 years, and 13 years for females. In the multivariate model, sex and age were found to be associated with early sexual initiation. Males were 7.30 times (95%CI=2.06-25.83) more likely to have experienced early sexual initiation than males. Those aged 15-17 were 8.31 times (95%CI=1.68-41.07) more likely to have experienced early sexual initiation than those who were 21-24 years, respectively.

Conclusions: A large proportion of the hill tribe youths have sexual experience, and one-tenth have had early sexual initiation before the age of 15 years. Specific knowledge on sexual education programs should be integrated into the high school study curriculum to protect early sexual initiation behaviors and safe sex, particularly in females. Family and community-based interventions should also be strongly recommended for the sexual education training implementation. The curriculum should focus on the impact of substance use and potentially early sexual initiation.

Keywords: Early sexual initiation, Influencing factors, Youth, Hill tribe

Introduction

Youths comprise an age group between 15-24 years old, as defined by the United Nations (UN) [1]. There is no exact definition for readiness to have sexual initiation. In addition to the use of a physical indicator of readiness to have sexual initiation, many other factors need to be considered, depending on individuals' backgrounds, such as socioeconomic status [2], mental mutuality [3], and social acceptance [4]. Having sexual initiation when one is not ready either physically or mentally will raise several problems in the future, such as sexually transmitted diseases [5, 6] and school dropout among young parents [7]. Ideally, sexual intercourse occurs when individuals reach both physical and psychological maturity [8]. However, there are several factors influencing early sexual initiation that arise before individuals reach maturity, particularly in young people, such as alcohol use [9], drug use [10], and social media use [11, 12]. The World Health Organization (WHO) defines early sexual initiation as having sexual intercourse before the age of 15 years ~~old~~; ~~these behaviors vary in different societies and frequently occur among youths [13]~~. Early sexual initiation could lead to several personal health and social problems in the future, which is a difficult issue to address. Youths, then, become vulnerable to early sexual initiation due to various influencing factors in different groups of populations.

In 2019, the WHO reported that people with low education and those living in poverty were more likely to have early sexual initiation than people who had a good education and high income [13]. The hill tribe people in Thailand lived in poor socioeconomic status [14, 15]. In

2019, six main groups were identified: the Akha, Lahu, Hmong, Yao, Karen, and Lisu; these groups had an overall population of approximately 4 million individuals in 16 provinces in Thailand [16]. Chiang Rai Province is located in the northernmost part of Thailand, bordered by Myanmar in the West, China in the North, and Laos in the East [17]. More than 350,000 hill tribe people live in 749 hill tribe villages in Chiang Rai Province [18]. The hill tribe people have their own lifestyle patterns [19], beliefs, perceptions [20], and acceptance of sexual intercourse [21].

Poor socioeconomic living conditions of the hill tribe people, including poor family and social relationships [19, 22], could lead the hill tribe youths to engage in early sexual initiation. Having early sexual initiation ~~the hill tribe youths who have been clearly defined as~~ vulnerable to sexually transmitted infections (STIs) [23], might be facing serious health problems from the infections and also unsafe abortion [24]. While ~~the family economic constraint of the hill tribe~~ [25], the hill tribe youths also face limited access to care, which will ~~cause them double the~~ suffering. In addition, a large proportion of hill tribe youths have been directly and indirectly forced to work in the cities [26], and the new living circumstances could lead them to have early sexual initiation. In addition, some cultures of the hill tribe accept exposure to sexual activity in their early life [18, 20]. Currently, there is no scientific information available regarding the issue. ~~Then~~, this study aimed to estimate the prevalence of and determine the factors associated with early sexual initiation ~~or~~ before the age of 15 years old among hill tribe youths. The results could be eventually used to develop policies and public health interventions.

Methods

Study design and study population

A cross-sectional study design was conducted to determine the factors associated with early sexual initiation among the hill tribe youths. The study design could provide the prevalence of and be able to determine the factors associated with early sexual initiation of the participants.

Study setting

The study was conducted in the hill tribe villages located in Chiang Rai province, Thailand. In 2018, There were 749 hill tribe villages in Chiang Rai, which broke down into 316 Lahu villages, 243 Akha villages, 63 Yao villages, 56 Hmong villages, 36 Karen villages, and 35 Lisu villages. In 2018, a total of 42,177 hill tribe families lived in Chiang Rai Province [27].

Study population

The study population was comprised of hill tribe youths aged 15-24 who lived in Chiang Rai Province. The target population consisted of six main groups: Akha, Lahu, Hmong, Yao, Karen, and Lisu.

Inclusion criteria were the hill tribe youths belonging to one of the six main hill tribes who lived in selected villages at the date of data collection. However, those who could not identify themselves as belonging to one of the six main tribes and unable to communicate in Thai were excluded from the study.

Study sample and sample size calculation

The study participants were 15-24 years old and were randomly selected from 30 hill tribe villages (5 villages for each tribe). The sample size was calculated based on the standard formula for a cross-sectional study design [28]; n = [Z2a/2 P(1-P)]/e2, where n = sample size required, Z = value from standard normal distribution corresponding to desired confidence level (Z = 1.96 for 95% CI), P = expected true proportion, and e = desired precision. Under the assumption of P=0.15 [29], and e=0.05, a total of 1,175 participants were required for the analysis. Adding 10% for any errors during the study, it needed 1,293 ~~samples in~~ the study.

Research instruments

A validated questionnaire was used to collect data from participants. The questionnaire was divided into four parts. In the first part, 13 questions were used to collect the general information of the participants, such as sex, age, and education. In the second part, 18 questions were used to collect information regarding health risk behaviors, such as drinking alcohol, smoking, and substance use. In the third part, 22 questions were used to collect information regarding the experience of early sexual initiation, ~~which is having their first sexual initiation~~ before the age of 15 years old, the age of their first sexual initiation, etc. In the last part, 20 questions were used to collect knowledge about safe sex and attitudes toward early sexual initiation behaviors and safe sex (S1 file Questionnaire).

Both the validity and the reliability of the questionnaire were determined before use. Three external experts (one public health expert, one epidemiologist, and one sexual and reproductive expert) in the field of sexual behavior were invited to validate the questions by item-objective congruence (IOC) techniques. Those questions that scored less than 0.5 were excluded, questions that scored 0.51-0.69 were revised as comments, and questions that scored ≥ 0.70 were included in the questionnaire without revision.

Afterward, the questionnaire was piloted on 15 individuals with characteristics similar to those of the study sample in Huan Hin Fon and Sun Ti Suk Village, Mae Chan District,

Chiang Rai Province. The purposes of doing a pilot were to detect the feasibility of using the questions for the participants, to check the proper sequence of the questions throughout the questionnaire, and to detect the reliability of the last part of the questionnaire, which had a Cronbach’s alpha of 0.71.

Steps of data collection

A cluster random method was used to select 30 villages, five villages in each tribe, from 749 hill tribe villages located in the study settings: 316 Lahu villages, 243 Akha villages, 63 Yao villages, 56 Hmong villages, 36 Karen villages, and 35 Lisu villages. The district government officers granted permission to access villages before the village headmen were contacted. Afterward, the village headmen were contacted five days before meeting them in the village. Information about the research project, including its objectives and procedures, was provided to the village headman.

A list of the youths in the selected villages was asked for and obtained after approval was given by the village headman. All youths in the village who met the study's criteria were invited to participate between November 2019 and March 2020. A further appointment was made to collect data from the target participants.

On the data collection date, all participants were provided information individually and asked for their cooperation by means of a signed informed consent form after a verbal explanation was provided. The participants completed the questionnaire independently in a private and confidential room in the village. For participants younger than 18 years old, an informed consent form was also obtained from their parents. The whole process of completing the questionnaire lasted for 25 minutes.

Variables

The dependent variable was the experience of having sexual initiation before the age of 15 years, which was classified into “yes” and “no”. Some key independent variables were categorized into proper forms. For instance, education was classified into four categories: no education, primary school, high school, and vocational school. Variables related to substance use, such as smoking, alcohol use, amphetamine use, etc., were classified into two categories each; “no” and “yes”. In the section on knowledge of safe sex (10 items), there were classified into three categories: poor (0-5 scored), moderate (6-8 scored), and good (9-10 scored) [23]. There were three responses in each item of 10 attitudes questions: agree, neutral, and disagree, with five positive questions, i.e., “HIV-infected person can work with other people,” and another five negative questions, i.e., “We should not use a bathroom with HIV-infected person.” In the positive questions, there were given flowing scores in the response: agree (3

scores), neutral (2 scores), and disagree (1 score). However, in the negative questions, there were given flowing scores in the response: agree (1 score), neutral (2 scores), and disagree (3 scores). Then, those who scored 0-15 were defined as low, those with 16-24 scores were moderate, and those with 25-30 were good attitudes towards early sexual initiation behaviors and safe sex.

Statistical analysis

All completed questionnaires were coded, double-entered, and kept in secured files. Data were computed and analyzed using SPSS version 24 (SPSS, Chicago, IL). Descriptive statistics were used to explain the characteristics of participants; means and SDs were used for the continuous variables, and proportions were used for the categorical variables.

Logistic regression was used to determine the association between independent and early sexual initiation among hill tribe youths at a significance level of a=0.05. At this step, we extracted only data that reported having had a sexual experience into the model. During the analysis, a variable was selected into the model one by one until the model was fitted. The pseudo R2 of Cox-Snell R2, Nagelkerke R2, and Hosmer-Lemshow chi-square were determined to fit the statistical model. Age, tribe, and education were controlled as confounder factors in the model before interpreting the final finding.

Ethical approval and consent to participate

The research proposal, including the tools and procedures of data collection, was approved by the Mae Fah Luang University Research Ethics Committee on Human Research (REH-60030). All participants provided written informed consent. Participants younger than 18 years old were required to ask their parents to sign an informed consent form before data collection began. Participants were compensated 200 baht ($6) each, which is declared in the process and application of the ethical approval system. All data collection processes were executed in a private and confidential room and performed in accordance with the relevant guidelines and regulations (Declaration of Helsinki).

Results

In total, 1,310 participants were recruited for the study. The majority were females (60.8%), ~~those~~ aged 15-17 years old (59.0%), members of the Akha tribe (34.0%) and Buddhists (56.2%). A large proportion of participants were single (94.3%), had graduated from high school (72.4%), and had no income (90.1%). Two hundred and eleven participants (16.1%) had experience working outside the village, and only 1.2% had experience working abroad (Table 1) (S2 file study data).

A total of 315 (24.0%) out of 1,310 reported that they had sexual experience, while 6.3% did not respond to the question. Among those who had sexual experience, 9.8% had their first sexual experience before the age of 15 years. Of those who had early sexual initiation, 87.0% were males (Table 1).

In the comparisons of general characteristics among early sexual initiation, non-early sexual initiation, never had sexual experience, and non-response, several characteristics were found statistically significant, except religion (Table 1).

Table 1 General characteristics of the participants

Characteristics	Total		Early sexual initiation				Never had sexual experience		No answer		χ^2	p-value
			Yes (≤14)		No (≥15)							
	n	%	n	%	n	%	n	%	n	%		
**Total**	**1,310**	**100.0**	**31**	**2.4**	**284**	**21.7**	**912**	**69.6**	**83**	**6.3**	**N/A**	**N/A**
**Sex**												
Male	514	39.2	27	5.3	121	23.5	323	62.3	43	8.4	42.21	<0.001*
Female	796	60.8	4	0.5	163	20.5	629	74.0	40	5.0		
**Age** (years)												
15-17	773	59.0	19	2.5	69	8.9	650	84.1	35	4.5	286.15	<0.001*
18-20	392	29.9	8	2.0	120	30.6	228	58.2	36	9.2		
21-24	145	11.1	4	2.8	95	65.5	34	23.4	12	8.3		
**Tribe**												
Akha	445	34.0	12	2.7	75	16.9	336	75.5	22	4.9	40.59	<0.001*
Lahu	204	15.6	4	2.0	65	31.9	120	58.8	15	7.4		
Hmong	212	16.2	5	2.4	48	22.6	145	68.4	14	6.6		
Yao	175	13.4	4	2.3	21	12.0	134	76.6	16	9.1		
Karen	158	12.1	4	2.5	41	25.9	106	67.1	7	4.4		
Lisu	116	8.9	2	1.7	34	29.3	71	61.2	9	7.8		
**Religion**												
Buddhism	736	56.2	16	2.2	169	23.0	496	67.4	55	7.5	9.25	0.131[a]
Christian	566	43.2	15	2.7	115	20.3	409	72.3	27	4.8		
Islam	8	0.6	0	0	0	0	7	87.5	1	12.5		
**Marital status**												
Single	1,235	94.3	28	2.3	223	18.1	910	79.7	74	6.0	178.53	<0.001[a]
Married	65	5.0	3	4.6	55	84.6	1	10.8	6	9.2		
Divorced	10	0.7	0	0.0	6	60.0	1	40.0	3	30.0		

* Significant level at α =0.05, [a] Fisher's exact test

Characteristics	Total		Early sexual initiation				Never had sexual experience		No answer		χ^2	p-value
			Yes (≤14)		No (>15)							
	n	%	n	%	n	%	n	%	n	%		
**Education**												
No education	45	3.4	2	4.4	17	37.8	18	40.0	8	17.8	139.13	<0.001*
Primary school	52	4.0	3	5.8	25	48.1	22	42.3	2	3.8		
High school	949	72.4	24	2.5	130	13.7	744	78.4	51	5.4		
Vocational school	246	18.8	1	0.4	105	42.7	122	49.6	18	7.3		
University	18	1.4	1	5.6	7	38.9	6	33.3	4	22.2		
**Occupation**												
Unemployed	111	8.5	5	4.5	54	48.6	47	46.8	5	4.5	251.56	<0.001*
Student	1,048	80.0	19	1.8	140	13.4	826	84.8	63	6.0		
Farmer	38	2.9	2	5.3	24	63.2	6	31.6	6	15.8		
Labor	113	8.7	5	4.4	66	58.4	33	37.2	9	8.0		
**Having regular income**												
No	1,180	90.1	24	2.0	213	18.1	864	73.2	79	6.7	102.35	<0.001*
Yes	130	9.9	7	5.4	71	54.6	48	36.9	4	3.1		
**Work experience outside village**												
No	1,099	83.9	20	1.8	203	18.5	108	51.2	11	5.2	53.78	<0.001*
Yes	211	16.1	11	5.2	81	38.4	804	73.2	72	6.6		
**Work experience abroad**												
No	1,294	98.8	29	2.2	273	21.1	910	70.3	82	6.3	26.88	<0.001 [a]
Yes	16	1.2	2	12.5	11	68.8	2	12.5	1	6.3		

* Significant level at α =0.05, [a] Fisher's exact test

Table 2 Substances use behaviors, knowledge of safe sex, and attitudes towards early sexual initiation behaviors and safe sex

Characteristics	Total		Early sexual initiation				Never had sexual experience		No answer		χ^2	p-value
			Yes (≤14)		No (>15)							
	n	%	n	%	n	%	n	%	n	%		
**Smoking**												
No	1,123	85.7	15	1.3	212	18.9	825	73.5	71	6.3	80.50	<0.001*
Yes	187	14.3	16	8.6	72	38.5	87	46.5	12	6.4		
**Alcohol use**												
No	1,019	77.8	13	1.3	162	15.9	777	76.3	67	6.6	123.18	<0.001*
Yes	291	22.2	18	6.2	122	41.9	135	46.4	16	5.5		
**Methamphetamine use**												
No	1,276	97.4	26	2.0	267	20.9	901	70.6	82	6.4	30.63	<0.001[a]
Yes	34	2.6	5	14.7	17	50.0	11	32.4	1	2.9		
**Heroin use**												
No	1,287	98.2	28	2.2	278	21.6	899	69.9	82	6.4	7.99	<0.036[a]
Yes	23	1.8	3	13.0	6	26.1	13	56.5	1	4.3		
**Crystal methamphetamine use**												
No	1,290	98.5	29	2.2	278	21.6	901	69.8	82	6.4	5.62	<0.088[a]
Yes	20	1.5	2	10.0	6	30.0	11	55.0	1	5.0		
**Level of knowledge of safe sex**												
Poor	1,045	79.8	25	2.4	191	18.3	758	72.5	71	6.8	37.01	<0.001[a]
Moderate	192	14.6	5	2.6	74	38.5	105	54.7	8	4.2		
Good	73	5.6	1	1.4	19	26.0	49	67.1	4	5.5		
**Level of attitudes towards early sexual initiation behaviors and safe sex**												
Poor	439	33.5	13	3.0	95	21.6	331	75.4	41	9.3	13.47	<0.036*
Moderate	574	43.8	13	2.3	118	20.6	443	77.2	26	4.5		
Good	297	22.7	5	1.7	71	23.9	221	74.4	16	5.4		

* Significant level at α =0.05, [a] Fisher's exact test

One-fourth of the participants used alcohol (22.2%), 14.3% smoked, and 2.6% used methamphetamine. A large proportion (79.8%) had a poor level of knowledge of safe sex, and 33.5% had a poor level of attitudes towards early sexual initiation behaviors and safe sex (Table 2).

Several substance use behaviors, knowledge of safe sex, and attitudes towards early sexual initiation behaviors and safe sex were found statistically significant among those who had early sexual initiation, non-early sexual initiation, never had sexual experience, and non-response (Table 2).

Table 3 Comparisons of sexual behavior between sexes

Characteristics	Total n (%)	Male n (%)	Female n (%)	χ^2	p-value
**Total**	**1,310**	**514**	**796**	**N/A**	**N/A**
**Having boyfriend or girlfriend**					
No	806 (61.5)	315 (61.3)	491 (61.7)	0.21	0.885
Yes	504 (38.5)	199 (38.7)	305 (38.3)		
**Number of boyfriends or girlfriends**					
One	484 (94.0)	184 (92.4)	300 (98.4)	10.99	0.001*
More than one	30 (6.0)	15 (7.6)	5 (1.6)		
**Having sexual experience**					
No	912 (69.6)	323 (62.8)	589 (74.0)	19.01	<0.001*
Yes	315 (24.1)	148 (47.0)	167 (53.0)		
No answer	83 (6.3)	43 (51.8)	40 (48.2)		
**Age at first sexual initiation** (years)					
≤14	31 (13.6)	27 (87.1)	4 (12.9)	24.59	<0.001*
≥ 15	284 (86.4)	121 (42.6)	163 (57.4)		
Mean overall = 16.7 (SD=2.0); Median= 16, Mean in males = 16.0 (SD=1.92); Mean in females = 17.4 (SD=1.90)					
**Having sexual experience with one nightstand**					
No	231(73.3)	94 (40.7)	137 (59.3)	13.76	<0.001*
Yes	84 (26.7)	54 (64.3)	30 (35.7)		
**Having sexual experience with sex worker one year prior**					
No	291(92.4)	131 (45.0)	160 (55.0)	5.93	0.015*
Yes	24 (7.6)	17 (70.8)	7 (29.2)		
**Having regular partner**					
No	191 (63.6)	107 (56.0)	84 (44.0)	15.9	<0.001*
Yes	124 (36.4)	41 (33.1)	83 (66.9)		
**Alcohol or drug use prior having sexual initiation**					
No	284 (76.4)	130 (45.8)	154 (54.2)	1.80	0.406
Sometime	18 (4.5)	10 (55.6)	8 (44.4)		
Yes	13 (19.1)	8 (61.5)	5 (38.5)		

* Significant level at α =0.05

Five hundred and four (38.5%) participants had boyfriends or girlfriends, and among them, 6.0 % had more than one boyfriend or girlfriend. One-fourth (24.1%) of the participants had experienced sexual initiation (28.8% of males and 20.9% of females). Among those who had sexual initiation, 13.6% had sexual initiation before 15 years of age. The overall age at first sexual initiation was 16.7 years. After further examination, it was found that the average age at first sexual initiation among males was 16.0 years, with the minimum age at first sexual initiation being 12 years, whereas the average age at first sexual initiation among females was 17.4 years, with the minimum age being 13 years (Table 3).

Eighty-four participants (26.7%) had experienced having sexual initiation with one-night stands (which is having sexual experience without knowing their partner), 7.6% had a sexual experience with a sex worker one year prior, and 19.1% regularly used alcohol or drugs prior to having sexual intercourse (Table 3).

In the comparisons of sexual behaviors between males and females, six factors were found to be statistical differences; number of boyfriends or girlfriends (p-value=0.001), having sexual experience (p-value<0.001), age at first sexual initiation (p-value<0.001), having sexual experience with one nightstand (p-value<0.001), having sexual experience with sex worker one year prior (p-value=0.050), and having regular partner (p-value<0.001) (Table 3).

Table 4 Univariable and multivariable analyses of factors associated with early sexual initiation among participants

Factors	Early sexual initiation		OR	95% CI	p-value	AOR	95% CI	p-value
	**Yes n (%)**	**No n (%)**						
**Total**	**31(9.8)**	**284 (90.2)**	**N/A**	**N/A**	**N/A**	**N/A**	**N/A**	**N/A**
**Sex**								
Male	27 (18.2)	121 (81.8)	9.09	3.10-26.67	<0.001*	7.30	2.06-25.83	0.002*
Female	4 (2.4)	163 (97.6)	1.00			1.00		
**Age** (years)								
15-17	19 (21.6)	69 (78.4)	6.54	2.13-20.08	0.001*	8.31	1.68-41.07	0.009*
18-20	8 (6.2)	120 (93.8)	4.13	1.71-9.93	0.002*	2.33	0.47-11.41	0.270
21-24	4 (4.0)	95 (96.0)	1.00			1.00		
**Tribe**								
Akha	12 (13.8)	75 (88.5)	1.00			1.00		
Lahu	4 (5.8)	65 (94.2)	0.38	0.11-1.25	0.112	0.57	0.11-2.78	0.493
Hmong	5 (9.4)	48 (90.6)	0.65	0.21-1.96	0.446	1.26	0.26-6.15	0.769
Yao	4 (16.0)	21 (84.0)	1.19	0.34-4.07	0.781	1.31	0.23-7.34	0.758
Karen	4 (8.9)	41 (91.1)	0.61	0.18-2.01	0.417	0.96	0.22-4.20	0.965
Lisu	2 (5.6)	34 (94.4)	0.36	0.07-1.73	0.206	0.40	0.05-3.08	0.380
**Religion**								
Buddhism	16 (8.6)	169 (91.4)	1.00			1.00		
Christianity	15 (11.5)	115 (88.5)	1.37	0.65-2.89	0.398	1.01	0.30-3.24	0.999

**Education**								
No education	2 (10.5)	17 (89.5)	1.00			1.00		
Primary school	3 (10.7)	25 (89.3)	1.02	0.15-6.76	0.984	1.77	0.12-25.24	0.673
≥High school	26 (9.7)	242 (90.3)	0.91	0.20-4.17	0.907	0.81	0.07-9.38	0.869
**Occupation**								
Student	19 (11.9)	140 (88.1)	1.00			1.00		
Unemployed	5 (8.5)	54 (91.5)	0.68	0.24-1.91	0.469	0.36	0.07-1.85	0.225
Farmer	2 (7.7)	24 (92.3)	0.61	0.13-2.80	0.529	0.55	0.05-5.82	0.625
Labor	5 (7.0)	66 (93.0)	0.55	0.20-1.56	0.266	0.38	0.06-2.25	0.290
**Having regular income**								
No	24 (10.1)	213 (89.9)	1.00			1.00		
Yes	7 (9.0)	71 (91.0)	1.14	0.47-2.76	0.767	1.41	0.29-6.79	0.669
**Smoking**								
No	15 (6.6)	212 (93.4)	1.00			1.00		
Yes	16 (18.2)	72 (81.8)	3.14	1.47-6.67	<0.003*	1.94	0.55-6.79	0.295
**Alcohol use**								
No	13 (7.4)	162 (92.6)	1.00			1.00		
Yes	18 (12.9)	122 (87.1)	1.83	0.86-3.89	0.112	0.48	0.15-1.55	0.222
**Methamphetamine use**								
No	26 (8.9)	267 (91.1)	1.00			1.00		
Yes	5 (22.7)	17 (77.3)	3.02	1.03-8.85	0.044*	1.83	0.30-11.05	0.508
**Heroin use**								
No	28 (9.2)	278 (90.8)	1.00			1.00		

Yes	3 (33.3)	6 (66.7)	4.96	1.17-20.93	0.029*	15.40	0.11-2044.62	0.273
**Crystal methamphetamine use**								
No	29 (9.4)	278 (90.6)	1.00			1.00		
Yes	2 (25.0)	6 (75.0)	3.19	0.61-16.56	0.166	0.12	0.01-22.58	0.431
**Work experience outside village**								
No	20 (9.0)	203 (91.0)	1.00			1.00		
Yes	11 (12.0)	81 (88.0)	1.37	0.63-3.00	0.420	2.21	0.73-6.66	0.156
**Work experience abroad**								
No	29 (13.4)	273 (90.4)	1.00			1.00		
Yes	2 (15.4)	11 (84.6)	1.71	0.36-8.10	0.498	4.03	0.42-38.18	0.224
**Level of knowledge of safe sex**								
Poor	25 (11.6)	191 (88.4)	2.48	0.31-19.38	0.385	1.90	0.11-32.08	0.656
Moderate	5 (6.3)	74 (93.7)	1.28	0.14-11.64	0.824	1.28	0.06-25.32	0.869
Good	1 (5.0)	19 (95.0)	1.00			1.00		
**Level of attitudes towards early sexual initiation behaviors and safe sex**								
Poor	13 (12.0)	95 (88.6)	1.94	0.66-5.70	0.226	0.76	0.18-3.17	0.715
Moderate	13 (9.9)	118 (90.1)	1.56	0.53-4.57	0.414	1.10	0.28-4.32	0.883
Good	5 (6.6)	71 (93.4)	1.00			1.00		

* Significance level at α =0.05

In the univariate model, five variables were found to be associated with early sexual initiation: sex, age, smoking, methamphetamine use, and heroin use. Males were 9.09 times (95%CI=3.10-26.67) more likely to have experienced early sexual initiation than females. Those aged 15–17 and 18–20 years were 6.54 times (95% CI = 2.13–20.08) and 4.13 times (95% CI = 1.71–9.93) more likely to have early sexual initiation than those aged 21–24 years, respectively. Those who smoked were 3.14 times (95% CI=1.47-6.67) more likely to have early sexual initiation than non-smoked. Those who used methamphetamine were 3.02 times (95% CI=1.03-8.85) more likely to have early sexual initiation than those who did not. Those who used heroin were 4.96 times (95% CI=1.17-20.93) more likely to have early sexual initiation than those who did not (Table 4).

In the multivariate model, sex and age were found to be associated with early sexual initiation. Males were 7.30 times (95%CI=2.06-25.83) more likely to have experienced early sexual initiation than females. Those aged 15–17 years were 8.31 (95% CI = 1.68–41.07) more likely to have early sexual initiation than those aged 21–24 years, respectively (Table 4).

Discussion

The hill tribe youths in Thailand used different substances, such as alcohol, tobacco, and amphetamines, and some of them used substances before having sexual initiation. One-fourth (24.0%) had sexual experience, while 6.3% did not respond whether they had experience or not. Among those who had sexual experience, 9.8% had their first sexual experience before the age of 15 years, and 87.1% were males. Females and younger age were detected as the key contributors to having early sexual initiation among the hill tribe youths in Thailand.

In our study, it was found that males (12 years old) had an earlier age of having first sexual initiation than females (13 years old). However, the overall age at first experiencing sexual initiation among the hill tribe youths was 16.7 years: 16.0 years old among males and 17.4 years old among females. In a study in South Africa among school children between grades 9 and 11 [30], it was reported that 50.4% of males and 30.1% of females had sexual experience. The median age at first sexual initiation for males and females was 16.5 years and 17.5 years, respectively. A study of Portuguese female students reported that the mean age of their first sexual initiation was 16.4 years old, which was younger than that of hill tribe women (16.7 years old); however, fewer people in the Portuguese study used substances (9.0%) before engaging in sexual initiation than did the hill tribe youths in Thailand (21.3%). A study in Ethiopia [31] reported that the mean age for sexual initiation among youths who had experienced sexual initiation was 15.4 years, while the corresponding age was 16.2 years

among the hill tribe youths in Thailand. A study in Ireland [32] reported that a greater proportion of girls (22.8%) than boys (13.4%) had sexual initiation prior to 14 years of age, in contrast to the findings for the hill tribe youths. A study conducted among the children who were attending school in the central region of Thailand [33] reported no sex-based differences in the proportion of early sexual experience among the youths (24.3% males and 24.0% females); however, 35.0% of the youths had more than one sexual partner. This rate is lower than that of the hill tribe youths having sexual initiation (30.4%), with a greater proportion of males than females having sexual initiation. In one more study [34] conducted among high school children aged 16-20 years living in rural northern areas of Thailand, it was found that 29.5% had experienced sexual initiation: 35% in males and 25.5% in females. Tangmunkongvorakul et al. [34] also reported that the average age of having first sexual initiation was 15.8 years: 15.9 years in males and 15.8 years in females. The hill tribe youths have a younger age of their first sexual initiation than Thai youths, which might be the culture of the hill tribe that needs to get married early to have a child for help in their family work. This is clearly supported by a study by Detpitukyon et al. stating that Lahu people need to marry early to support their family [35].

Early sexual behavior among the hill tribe was found in a larger proportion of males than females. This coincides with a longitudinal analysis conducted among Latin American youths, which reported that males had greater odds of having early sexual initiation than females [36]. Gazendam, et al. [37] also reported that being boys was a greater chance to have early sexual initiation than girls in Canada. A study in Nigeria [38] supported that being male was detected as the factor associated with having early sexual initiation among high school students. A study among vocational students in northern Thailand reported that males were detected as one of the factors associated with having early sexual initiation, particularly those who used alcohol [39]. The ~~male gender dominant~~ [35] and the culture of having early marriage among the hill tribe could lead them to have early sexual initiation.

Our study found that the younger age group of the hill tribe youths have a greater chance to have early sexual initiation than those older youths significantly. Our study has almost 10-year intervals between 15 and 24 years of the youths, then with a wide interval range of age; younger ages might be exposed to new technology and mass media of pornographic materials and seeking partners, which leads them to have early sexual initiation. Lay et al. [40] reported that younger youths, both boys and girls, were associated with early sexual initiation among Brazilian adolescents.

Several substances use behaviors were associated with early sexual initiation among the hill tribe youths including smoking, methamphetamine use, and heroin use. Exposing Substances could lead to uncontrol individuals' emotions, particularly young age people, and having sexual intercourse eventually. A systematic review of cohort studies reported that youths' substance use behaviors were one of the strong predictors of early sexual initiation [41]. A study conducted in the United States also reported that substance use increased early sexual initiation [42]. Substance use was a significant predictor of early sexual initiation among young people in Brazil [43] and Kenya [44].

In our study, it was found that 83 participants (6.3%) did not respond to the question asking about sexual initiation experience. Tamorpark et al. [45] reported that asking a sensitive question, particularly sexual behaviors and substance use, by audio computer-assisted self-interview gained a higher accuracy than a paper-based questionnaire. With the circumstance of the hill tribe culture that they are not familiar with discussing sexual behaviors with anyone, then this is not an overestimate for the researchers. In several traditional cultures, especially marriage in early life [35] and parenting styles in keeping sexual expression uncommon discussion among family members [20], young people tend to learn sexual education from the media. Moreover, several hill tribe youths move to the city for education or work, the opportunity to be exposed to early sexual initiation at an early age. The major driving factors that contribute to the hill tribe youths' exposure to early sexual initiation are poverty and limited literacy in both their parents and themselves.

A few limitations were found during the study. Many questions on the study protocol inquire about sexual behaviors, while some tribes, such as Lahu women, do not commonly talk about sexual behavior in their culture, particularly in public areas. Therefore, the findings on sexual behaviors could be underestimated in some aspects; however, the researchers explained to the participants as thoroughly as possible that their information would remain private and confidential and that it could not be used to identify the participants later on. During the process of data collection, all essential information, including concerns regarding privacy and confidentiality, was carefully explained to all participants to increase their willingness to answer the questions truthfully. The participants cooperated well with this aspect of the study. One more point, the number of participants in each tribe was different which could lead to the quality of the generalization of the findings to the specific tribe. Finally, a few participants did not clearly understand the context of some questions due to limited literacy. To address this issue, the researchers personally explained the meaning and content of confusing items. The study showed various strengths, such as the concept of investigating the very sensitive issue of

early sexual initiation among the marginalized population among six main hill tribes and the large number of participants recruited into the study.

Conclusion

Hill tribe youths are facing a problem of early sexual initiation, particularly male youths. Several substances are contributing to early sexual initiation among the hill tribe youths. Therefore, the interventions to reduce early sexual initiation behavior among the hill tribe youths should be focused on male gender and substance use. Understanding the context social norms and culture of sexual activity and the ability to discuss the issue at the family level are also critical points. ~~The intervention of~~ sexual education among family members, community-oriented, and extracurricular to add to the formal education in Thailand should be implemented.

Supplementary information

S1 file 1. Questionnaire used in the study.

S2 file 2. Study data

Acknowledgements

We would like to thank all village headmen for their kind support in accessing the participants. We also would like to thank all participants for providing essential information in the study.

Abbreviations

CI	Confident interval
HIV	Human immunodeficiency virus
IOC	Item-objective congruence
UN	United Nations
WHO	World Health Organization

Declarations

Author Contributions

Conceptualization: Tawatchai Apidechkul, Ratipark Tamornpark

Data curation: Tawatchai Apidechkul, Ratipark Tamornpark, Chalitar Chomchoei, Panupong Upala, Fartima Yeemard.

Formal analysis: Tawatchai Apidechkul, Ratipark Tamornpark

Funding acquisition: Tawatchai Apidechkul.

Investigation: Tawatchai Apidechkul, Ratipark Tamornpark, Chalitar Chomchoei, Panupong Upala, Fartima Yeemard.

Methodology: Tawatchai Apidechkul, Ratipark Tamornpark

Project administration: Tawatchai Apidechkul.

Supervision: Tawatchai Apidechkul, Ratipark Tamornpark

Writing – original draft: Tawatchai Apidechkul, Ratipark Tamornpark, Chalitar Chomchoei, Panupong Upala, Fartima Yeemard.

Writing – review & editing: Tawatchai Apidechkul, Ratipark Tamornpark, Chalitar Chomchoei, Panupong Upala, Fartima Yeemard.

References

1. United Nations (UN). World youth report 2018: youth and the 2030 agenda for sustainable development, UN, New York. 2018. doi: https://doi.org/10.18356/0c6f53e0-en.

2. Anguzu G, Flynn A, Musaazi J, Kasirye R, Atuhaire LK, Kiragga AN, et al. Relationship between socioeconomic status and risk of sexually transmitted infections in Uganda: multilevel analysis of a nationally representative survey. Int J STD AIDS. 2019; 30(3): 284-291. DOI: https://doi.org/10.1177/0956462418804115. Epub 2018 Nov 11.

3. World Health Organization (WHO). Responding to children and adolescents who have been sexually abused: WHO clinical guidelines. Geneva: World Health Organization; 2017. Available from: https://apps.who.int/iris/bitstream/handle/10665/259270/9789241550147-eng.pdf;jsessionid=C5DCAF1BDBE1C5E0A3AD4F7F8331EC12?sequence=1

4. Wesche R, Kreager DA, Feinberg ME. Peer acceptance and sexual behaviors from adolescence to young adulthood. J Youth Adolescence. 2019; 48: 996. 1-20. DOI: https://doi.org/10.1007/s10964-019-00991-7

5. Epstein M, Manhart LE, Hill KG, Bailey JA, Hawkins JD, Haggerty KP, et al. Understanding the link between early sexual initiation and later sexually transmitted infection: test and replication in two longitudinal studies. J Adolesc Health. 2014; 54(4): 435-441

6. Speizer I, Okigbo CC. Determinants of sexual activities and pregnancy among unmarried young women in urban Kenya: a cross-sectional study. PLoS One. 2015; 10(6): e0129286.

7. Glynn JR, Sunny BS, DeStavola B, Dube A, Chihana M, Price AJ. Early school failure predicts teenage pregnancy and married: a large population-based cohort study in northern Malawi. PLos One. 2018; 13(5): e0196041.

8. Yaya S, Bishwajit G. Age at first sexual intercourse and multiple sexual partnerships among women in Nigeria: a cross-sectional analysis. Front Med (Lausanne). 2018; 5:171. DOI: https://doi.org/10.3389/fmed.2018.00171.

9. Brown JL, Gause NK, Northern N. The Association between alcohol and sexual risk behaviors among college students: a review. Curr Addict Rep. 2016; 3(4): 349-355. DOI: https://doi.org/10.1007/s40429-016-0125-8.
10. Yazdi V, Mehrolhassani M, Zolala F, Haghdoost A, Oroomiei N. Determinants of risky sexual practice, drug abuse and alcohol consumption in adolescents in Iran: a systematic literature review. BMC Reprod Health. 2019; 16: 115-125.
11. Landry M, Turner M, Vyas A, Wood S. Social media and sexual behavior among adolescents: Is there a link? JMIR Public Health Surveill 2017;3(2). DOI: https://doi.org/10.2196/publichealth.7149.
12. Merrilla RA, Liang X. Associations between adolescent media use, mental health, and risky sexual behaviors. Child. Youth Serv Rev 2019; 103: 1-9. DOI: https://doi.org/10.1016/j.childyouth.2019.05.022
13. World Health Organization (WHO). High rates of unintended pregnancies linked to gaps of family planning. Available from: https://www.who.int/news-room/detail/25-10-2019-high-rates-of-unintended-pregnancies-linked-to-gaps-in-family-planning-services-new-who-study.
14. Thutsanti P, Apidechkul T, Inta C, Upala P, Tamornpark R. Adaptation to Thai citizenship: a case study of Thai-Chinese Yunnan, Mae Fah Laung District, Chiang Rai, Thailand. J Health Sci Altern Med. 2019; 1(1): 8-13.
15. Chomchoei C, Apidechkul T, Wongnuch P, Tamornpark R, Upala P, Nongkhai MPN. Perceived factors influencing the initiation of methamphetamine use among Akha and Lahu youths: a qualitative approach. BMC Public Health. 2019;19(847): 1-11. DOI: https://doi.org/10.1186/s12889-019-7226-y.
16. Princess Maha Chakri Siridhorn Anthropology Center. Hill tribe. Available from: http:// www.sac.or.th/main/index.php. Assessed 15 Nov 2019
17. Department of Mineral Resources. Geology of Thailand. Ministry of Natural Resources and Environment. 2016. Available from: http://www.dmr.go.th/main.php?filename=GeoThai_En
18. Apidechkul T, Wongnuch P, Sittisarn S and Ruanjai T. Health situation of Akha hill tribe in Chiang Rai Province, Thailand. J Pub Health Dev. 2016; 14(1):77-97.
19. Jakae O, Tanpichai P, Jaiaree A. Construction of social space in Thai hill tribe ethnic groups in Kamphaeng Phet province. Kasetsart Journal of Social Sciences. 2018. 1-7. DOI: https://doi.org/10.1016/j.kjss.2018.07.019
20. Singkorn O, Apidechkul T, Putsa B, Detpetukyon S, Sunsern R, Thutsanti P, et al. Factor associated with alcohol use among Lahu and Akha hill tribe youths, northern

Thailand. Subst Abuse Treat Prev Policy. 2019; 14(5); 1-14. doi: 10.1186/s13011-019-0193-6.

21. Aurpibul L, Tangmunkongvorakul A, Musumari PM, Srithanaviboonchai K, Tarnkehard S. Patterns of sexual behavior in lowland Thai youth and ethnic minorities attending high school in rural Chiang Mai, Thailand. PLoS One. 2016; 11(12). 1-17. DOI: 10.1371/journal.pone.0165866.

22.Thomas PA, Liu H, Umberson D. Family relationships and well-being. Innov Aging. 2017; 1(3).1-11. DOI: 10.1093/geroni/igx025.

23. Apidechkul T. Sexual behaviors and seroprevalence of HIV, HBV, and HCV among hill tribe youths of Northern Thailand. BMC Public Health. 2019; 19(1101). 1-17. DOI: https://doi.org/10.1186/s12889-019-7459-9

24. Chunuan S, Kosunvanna S, Sripotchanart W, Lawantrakul J, Lawantrakul J, Pattrapakdikul U, et al. Characteristics of abortion in southern Thailand. Pacific Rim Int J Nurse Res. 2012; 16(2): 97-112.

25. Apidechkul T, Laingoen O, Suwannaporn S. Inequity in accessing health care service in Thailand in 2015: a case study of the hill tribe people in Mae Fah Luang District, Chiang Rai, Thailand. J Health Res. 2016; 30 (1): 67-71.

26. Apidechkul T. Epidemiology of the hill tribe HIV/AIDS populations, Thailand. J Med Assoc Thai. 2016; 99(6): 702-710.

27. The Hill tribe Welfare and Development Center, Chiang Rai Province. Hill tribe population. In: The hill tribe welfare and development center. Chiang Rai: Ministry of Social Development and Human Security; 2018. p. 17–25.

28. Pouhoseingholi MA, Vahedi M, Rahimzadeh. Sample size calculation in medical studies. Gastroenterol Hepatol Bed Bench. 2013; 6(1): 14-17.

29. Franca MT, Frio GS. Factors associated with family, school and behavioral characteristics on sexual initiation: a gender analysis for Brazilian adolescents. PlosOne. 2018; 13(12). 1-16. DOI: 10.1371/journal.pone.0208542

30. Amoateng AY, Kaule-Sabiti I. Biosocial correlates of age at first sexual intercourse: the case of grade 9 and grade 11 pupils in the northwest province of South Africa. Journal of Biosocial Science. 2014; 1-17. DOI: 10.1017/S0021932014000492.

31. Girmay A, Mariye T. Risky sexual behavior practice and associated factors among secondary and preparatory school students of Aksum town, northern Ethiopia, 2018. BMC Res Nores. 2019; 12(698): 1-7. DOI: 10.1186/s13104-019-4714-1.

32. Young H, Burk L, Gabhainn SN. Sexual intercourse, age of initiation and contraception among adolescents in Ireland: findings from the health behavior in school-aged children (HBSC) Ireland. BMC Public Health. 2018; 18(362). 1-17. DOI: 10.1186/s12889-018-5217-z.

33. Srijaiwong S, Sindhu S, Ratinthorn A, Viwatwongkasem C. Factors influencing sexual behaviors among Thai adolescents. Journal of Population and Social Studies. 2017; 25(3): 171-193.

34. Tangmunkongvorakul A, Srithanaviboonchai K, Guptarak M, Wichajarn M, Yungyuankul S, Khampan R, et al. Attitudes and behavior among rural Thai adolescents regarding sexual intercourse. Southeast Asian J Trop Med Public Health. 2014; 45(6): 1437-1447.

35. Detpitukyon S, Apidechkul T, Sunsern R, Anuwatnonthakate A, Singhhorn O, Putsa B, et al. Pattern and perception of alcohol drinking among the Lahu people, northern Thailand. Journal of Health Research. 2018; 32(3): 185-197.

36. Cueto S, Leon J. early sexual initiation among adolescents: a longitudinal analysis for 15-year-olds in Peru. Interamerican Journal of Psychology. 2016; 50(2): 186-203.

37. Gazendam N, Cleverley K, King N, Pickett W, Phillip SP. Individual and social determinants of early sexual activity: a study of gender-based differences using the 2018 Canadian Health Behavior in school-aged children study (HBSC). PlosOne. 2020; 15(9): 1-13. DOI: 10.1371/journal.pone.0238515.

38. Durowade KA, Babatunde OA, Omokanye LO, Elegbede OE, Ayodele LM, Adewoye KR, et al. Early sexual debut: prevalence and risk factors among secondary school students in Ido-ekit, Ekiti state, South-West Nigeria. Afr Health Sci. 2017; 17(3): 614-622.

39. Liu A, Kilmarx P, Jenkins RA, Manopaiboon C, Mock PA, Jeeyapunt S, et al. Sexual initiation, substance use, and sexual behavior and knowledge among vocational students in northern Thailand. International Family Planning Perspective. 2006; 32(3): 126-135.

40. Lay AA, Fujimori E, Duarte LS, Borges AL. Prevalence and correlates of early sexual initiation among Brazilian adolescents. Plosone. 2021; 16(12).: e0260815.

41. Reis LF, Surkan PJ, Atkins K, Garcia-Cerde R, Sanchez ZM. Risk factors for early sexual intercourse in adolescence: a systematic review of cohort studies. Child Psychiatry and Human Development. 2023. DOI:10.1007/s10578-023-01519-8.

42. Clark DA, Donnellan MB, Durbin CE, Nuttall AK, Hicks BM, Robins RW. Sex, drugs, and early emerging risk: examining the association between sexual debut and substance use across adolescence. Ploseone. 2020;15(2): e0228432.

43. Reis LF, Surkan PJ, Valente JY, Bertolla MH, Sanchez ZM. Factors associated with early sexual initiation and unsafe sex in adolescents: substance use and parenting style. Journal of Adolescence.2020; 79: 128-35.

44. Sing'oei V, Owuoth JK, Otieno J, Yates A, Aadalagu B, Smith HJ, et al. early sexual debut is associated with drug and decreased educational attainment among males and females in Kisumu country, Kenya. Reproductive Health. 2023; 20(11). DOI: 10.1186/s12978-023-01639-3.

45. Tamornpark R, Mulikaburt T, Adplong A, Upala P, Chomchoie C, Yeemard F. Effectiveness of the hill tribe audio computer assisted self interview versus self administration questionnaire to assess sexual behaviors among the hill tribe youths. 2021;3(3): 22-29

Quetaionniare_ENG

Click here to access/download
Supporting Information
Questionniare_ENG.pdf

Click here to access/download
Supporting Information
Questionniare_Thai.pdf

Study data

Click here to access/download
Supporting Information
Data_sexual.xlsx

Influencing factors early sexual initiation among hill tribe youths in Chiang Rai Province, northern Thailand: A community-based cross-sectional study

Ratipark Tamornpark[1,2], Tawatchai Apidechkul[1, 2, *], Panupong Upala[1,2], Chalitar Chomchoei[1,2], Fartima Yeemard[1]

[1]Center of Excellence for The Hill tribe Health Research, Mae Fah Luang University, Chiang Rai, Thailand

[2]School of Health Science, Mae Fah Luang University, Chiang Rai, Thailand

Authors' email

Ratipark Tamornpark email: ratipark.tam@mfu.ac.th

Tawatchai Apidechkul email: Tawatchai.api@mfu.ac.th

Panupong Upala email: panupong.upa@mfu.ac.th

Chalitar Chomchoei email: chalitar.cho@mfu.ac.th

Fartima Yeemard email: fartima.far052@gmail.com

[*]Correspondence: Tawatchai.api@mfu.ac.th

Abstract

Background: Early sexual initiation, before the age of 15 years, prior to full physical and mental maturity~~ could lead to several problems~~, could lead to several problems, including sexually transmitted diseases. The study aimed to estimate the prevalence of and determine the factors associated with early sexual initiation among hill tribe youths in Thailand.

Methods: A cross-sectional study was applied to gather information using a validated questionnaire from the hill tribe youths aged 15-24 years living in 30 selected hill tribe villages in Chiang Rai Province, Thailand. Data were obtained by self-report in a private and confidential room. Chi-square tests and logistic regression were used to detect the associations between variables at a significance level of α=0.05.

Results: A total of 1,310 hill tribe youths participated in the study: 60.8% were females, 59.0% were aged 15-17 years, 34.0% were Akha, and 90.1% had no income. Substance use~~, ~~: 22.2% used alcohol, 14.3% smoked, and 2.6% used methamphetamine. A large proportion (79.8%) had poor knowledge of safe sex, and 33.5% had poor attitudes towards early sexual behaviors and safe sex. One-third (30.4%) of the participants had sexual experience~~d~~: 37.2% of males and 26.0% of females. One-fourth (24.0%) had sexual experience. Among those who had sexual experience~~d~~, 9.8% had their first sexual initiation before 15 years. The overall age at

first sexual initiation was 16.7 years. The minimum age of having first sexual initiation for males was 12 years, and 13 years for females. In the multivariate model, sex and age were found to be associated with early sexual initiation. Males were 7.30 times (95%CI=2.06-25.83) more likely to have experienced early sexual initiation than males. Those aged 15-17 were 8.31 times (95%CI=1.68-41.07) more likely to have experienced early sexual initiation than those who were 21-24 years, respectively.

Conclusions: A large proportion of the hill tribe youths have ~~experienced experience~~sexual experience, and one-tenth have had early sexual initiation before the age of 15 years. ~~A s~~Specific ~~training~~ knowledge on sexual education programs should be integrated into the high school study curriculum to protect early sexual initiation behaviors and safe sex, particularly in females. Family and community-based interventions should also be strongly recommended for the sexual education training implementation. The curriculum should focus on the impact of substance use and potentially early sexual initiation.

Keywords: Early sexual initiation, Influencing factors, Youth, Hill tribe

Introduction

Youths comprise an age group between 15-24 years old, as defined by the United Nations (UN) [1]. There is no exact definition for readiness to have sexual initiation. In addition to the use of a physical indicator of readiness to have sexual initiation, many other factors need to be considered, depending on individuals' backgrounds, such as socioeconomic status [2], mental mutuality [3], and social acceptance [4]. Having sexual initiation when one is not ready either physically or mentally will raise several problems in the future, such as sexually transmitted diseases [5, 6] and school dropout among young parents [7]. Ideally, sexual intercourse occurs when individuals reach both physical and psychological maturity [8]. However, there are several factors influencing early sexual initiation that arise before individuals reach maturity, particularly in young people, such as alcohol use [9], drug use [10], and social media use [11, 12]. The World Health Organization (WHO) defines early sexual initiation as having sexual intercourse before the age of 15 years old; these behaviors vary in different societies and frequently occur among youths [13]. Early sexual initiation could lead to several personal health and social problems in the future, which is a difficult issue to address. Youths, then, become vulnerable to early sexual initiation due to various influencing factors in different groups of populations.

In 2019, the WHO reported that people with low education and those living in poverty were more likely to have early sexual initiation than people who had a good education and high income [13]. The hill tribe people in Thailand lived in poor socioeconomic status [14, 15]. In

2019, six main groups were identified: the Akha, Lahu, Hmong, Yao, Karen, and Lisu; these groups had an overall population of approximately 4 million individuals in 16 provinces in Thailand [16]. Chiang Rai Province is located in the northernmost part of Thailand, bordered by Myanmar in the West, China in the North, and Laos in the East [17]. More than 350,000 hill tribe people live in 749 hill tribe villages in Chiang Rai Province [18]. The hill tribe people have their own lifestyle patterns [19], beliefs, perceptions [20], and acceptance of sexual intercourse [21].

Poor socioeconomic living conditions of the hill tribe people, including poor family and social relationships [19, 22], could lead the hill tribe youths to engage in early sexual initiation. Having early sexual initiation the hill tribe youths who have been clearly defined as vulnerable to sexually transmitted infections (STIs) [23], might be facing serious health problems from the infections and also unsafe abortion [24]. While the family economic constraint of the hill tribe [25], the hill tribe youths also face limited access to care, which will cause them double the suffering. In addition, a large proportion of hill tribe youths have been directly and indirectly forced to work in the cities [26], and the new living circumstances could lead them to have early sexual initiation. In addition, some cultures of the hill tribe accept exposure to sexual activity in their early life [18, 20]. Currently, there is no scientific information available regarding the issue. Then, this study aimed to estimate the prevalence of and determine the factors associated with early sexual initiation or before the age of 15 years old among hill tribe youths. The results could be eventually used to develop policies and public health interventions.

Methods

Study design and study population

A cross-sectional study design was conducted to determine the factors associated with early sexual initiation among the hill tribe youths. The study design could provide the prevalence of and be able to determine the factors associated with early sexual initiation of the participants.

Study setting

The study was conducted in the hill tribe villages located in Chiang Rai province, Thailand. In 2018, There were 749 hill tribe villages in Chiang Rai, which broke down into 316 Lahu villages, 243 Akha villages, 63 Yao villages, 56 Hmong villages, 36 Karen villages, and 35 Lisu villages. In 2018, a total of 42,177 hill tribe families lived in Chiang Rai Province [27].

Study population

The study population was comprised of hill tribe youths aged 15-24 who lived in Chiang Rai Province. The target population consisted of six main groups: Akha, Lahu, Hmong, Yao, Karen, and Lisu.

Inclusion criteria were the hill tribe youths belonging to one of the six main hill tribes who lived in selected villages at the date of data collection. However, those who could not identify themselves as belonging to one of the six main tribes and unable to communicate in Thai were excluded from the study.

Study sample and sample size calculation

The study participants were 15-24 years old and were randomly selected from 30 hill tribe villages (5 villages for each tribe). The sample size was calculated based on the standard formula for a cross-sectional study design [28]; n = [Z2a/2 P(1-P)]/e2, where n = sample size required, Z = value from standard normal distribution corresponding to desired confidence level (Z = 1.96 for 95% CI), P = expected true proportion, and e = desired precision. Under the assumption of P=0.15 [29], and e=0.05, a total of 1,175 participants were required for the analysis. Adding 10% for any errors during the study, it needed 1,293 samples in the study.

Research instruments

A validated questionnaire was used to collect data from participants. The questionnaire was divided into four parts. In the first part, 13 questions were used to collect the general information of the participants, such as sex, age, and education. In the second part, 18 questions were used to collect information regarding health risk behaviors, such as drinking alcohol, smoking, and substance use. In the third part, 22 questions were used to collect information regarding the experience of early sexual initiation, which is having their first sexual initiation before the age of 15 years old, the age of their first sexual initiation, etc. In the last part, 20 questions were used to collect knowledge about safe sex and attitudes toward early sexual initiation behaviors and safe sex (S1 file Questionnaire).

Both the validity and the reliability of the questionnaire were determined before use. Three external experts (one public health expert, one epidemiologist, and one sexual and reproductive expert) in the field of sexual behavior were invited to validate the questions by item-objective congruence (IOC) techniques. Those questions that scored less than 0.5 were excluded, questions that scored 0.51-0.69 were revised as comments, and questions that scored ≥ 0.70 were included in the questionnaire without revision.

Afterward, the questionnaire was piloted on 15 individuals with characteristics similar to those of the study sample in Huan Hin Fon and Sun Ti Suk Village, ~~the~~ Mae Chan District,

Chiang Rai Province. The purposes of doing a pilot were to detect the feasibility of using the questions for the participants, to check the proper sequence of the questions throughout the questionnaire, and to detect the reliability of the last part of the questionnaire, which had a Cronbach's alpha of 0.71.

Steps of data collection

A cluster random method was used to select 30 villages, five villages in each tribe, from 749 hill tribe villages located in the study settings: 316 Lahu villages, 243 Akha villages, 63 Yao villages, 56 Hmong villages, 36 Karen villages, and 35 Lisu villages. The district government officers granted permission to access villages before the village headmen were contacted. Afterward, the village headmen were contacted five days before meeting them in the village. Information about the research project, including its objectives and procedures, was provided to the village headman.

A list of the youths in the selected villages was asked for and obtained after approval was given by the village headman. All youths in the village who met the study's criteria were invited to participate between November 2019 and March 2020. A further appointment was made to collect data from the target participants.

On the data collection date, all participants were provided information individually and asked for their cooperation by means of a signed informed consent form after a verbal explanation was provided. The participants completed the questionnaire independently in a private and confidential room in the village. For participants younger than 18 years old, an informed consent form was also obtained from their parents. The whole process of completing the questionnaire lasted for 25 minutes.

Variables

The dependent variable was the experience of having sexual initiation before the age of 15 years, which was classified into "yes" and "no". Some key independent variables were categorized into proper forms. For instance, education was classified into four categories: no education, primary school, high school, and vocational school. Variables related to substance use, such as smoking, alcohol use, amphetamine use, etc., were classified into two categories each; "no" and "yes". In the section on knowledge of safe sex (10 items), there were classified into three categories: poor (0-5 scored), moderate (6-8 scored), and good (9-10 scored) [23]. There were three responses in each item of 10 attitudes questions: agree, neutral, and disagree, with five positive questions, i.e., "HIV-infected person can work with other people," and another five negative questions, i.e., "We should not use a bathroom with HIV-infected person." In the positive questions, there were given flowing scores in the response: agree (3

scores), neutral (2 scores), and disagree (1 score). However, in the negative questions, there were given flowing scores in the response: agree (1 score), neutral (2 scores), and disagree (3 scores). Then, those who scored 0-15 were defined as low, those with 16-24 scores were moderate, and those with 25-30 were good attitudes towards early sexual initiation behaviors and safe sex.

Statistical analysis

All completed questionnaires were coded, double-entered, and kept in secured files. Data were computed and analyzed using SPSS version 24 (SPSS, Chicago, IL). Descriptive statistics were used to explain the characteristics of participants; means and SDs were used for the continuous variables, and proportions were used for the categorical variables.

Logistic regression was used to determine the association between independent and early sexual initiation among hill tribe youths at a significance level of a=0.05. At this step, we extracted only data that reported having had a sexual experience into the model. During the analysis, a variable was selected into the model one by one until the model was fitted. The pseudo R2 of Cox-Snell R2, Nagelkerke R2, and Hosmer-Lemshow chi-square were determined to fit the statistical model. Age, tribe, and education were controlled as confounder factors in the model before interpreting the final finding.

Ethical approval and consent to participate

The research proposal, including the tools and procedures of data collection, was approved by the Mae Fah Luang University Research Ethics Committee on Human Research (REH-60030). All participants provided written informed consent. Participants younger than 18 years old were required to ask their parents to sign an informed consent form before data collection began. Participants were compensated 200 baht ($6) each, which is declared in the process and application of the ethical approval system. All data collection processes were executed in a private and confidential room and performed in accordance with the relevant guidelines and regulations (Declaration of Helsinki).

Results

In total, 1,310 participants were recruited for the study. The majority were females (60.8%), those aged 15-17 years old (59.0%), members of the Akha tribe (34.0%) and Buddhists (56.2%). A large proportion of participants were single (94.3%), had graduated from high school (72.4%), and had no income (90.1%). Two hundred and eleven participants (16.1%) had experience working outside the village, and only 1.2% had experience working abroad (Table 1) (S2 file study data).

A total of 315 (24.0%) out of 1,310 reported that ~~had sexual experience, while 6.3% did not response~~they had sexual experience, while 6.3% did not respond to the question. Among those who had sexual~~ experienced; 9.8% were had their first sexual experience befor~~ experience, 9.8% had their first sexual experience before the age of 15 years. ~~T~~Of those who had early sexual initiation, 87.0% were males (Table 1).

In the comparisons of general characteristic~~ among early sexual initiation, non-early sexual initiation, never had sexual experience, and non-response, several characteristics were found statistically significant, except,~~s among early sexual initiation, non-early sexual initiation, never had sexual experience, and non-response, several characteristics were found statistically significant, except religion (Table 1).

Table 1 General characteristics of the participants

Characteristics	Total		Early sexual initiation				Never had sexual experience		No answer		χ^2	p-value
			Yes (≤14)		No (≥15)							
	n	%	n	%	n	%	n	%	n	%		
**Total**	**1,310**	**100.0**	**31**	**2.4**	**284**	**21.7**	**912**	**69.6**	**83**	**6.3**	**N/A**	**N/A**
**Sex**												
Male	514	39.2	27	5.3	121	23.5	323	62.3	43	8.4	42.21	<0.001*
Female	796	60.8	4	0.5	163	20.5	629	74.0	40	5.0		
**Age** (years)												
15-17	773	59.0	19	2.5	69	8.9	650	84.1	35	4.5	286.15	<0.001*
18-20	392	29.9	8	2.0	120	30.6	228	58.2	36	9.2		
21-24	145	11.1	4	2.8	95	65.5	34	23.4	12	8.3		
**Tribe**												
Akha	445	34.0	12	2.7	75	16.9	336	75.5	22	4.9	40.59	<0.001*
Lahu	204	15.6	4	2.0	65	31.9	120	58.8	15	7.4		
Hmong	212	16.2	5	2.4	48	22.6	145	68.4	14	6.6		
Yao	175	13.4	4	2.3	21	12.0	134	76.6	16	9.1		
Karen	158	12.1	4	2.5	41	25.9	106	67.1	7	4.4		
Lisu	116	8.9	2	1.7	34	29.3	71	61.2	9	7.8		
**Religion**												
Buddhism	736	56.2	16	2.2	169	23.0	496	67.4	55	7.5	9.25	0.131[a]
Christian	566	43.2	15	2.7	115	20.3	409	72.3	27	4.8		
Islam	8	0.6	0	0	0	0	7	87.5	1	12.5		
**Marital status**												
Single	1,235	94.3	28	2.3	223	18.1	910	79.7	74	6.0	178.53	<0.001[a]
Married	65	5.0	3	4.6	55	84.6	1	10.8	6	9.2		
Divorced	10	0.7	0	0.0	6	60.0	1	40.0	3	30.0		

* Significant level at α =0.05, [a] Fisher's exact test

Characteristics	Total		Early sexual initiation				Never had sexual experience		No answer		χ^2	p-value
			Yes (≤14)		No (>15)							
	n	%	n	%	n	%	n	%	n	%		
**Education**												
No education	45	3.4	2	4.4	17	37.8	18	40.0	8	17.8	139.13	<0.001*
Primary school	52	4.0	3	5.8	25	48.1	22	42.3	2	3.8		
High school	949	72.4	24	2.5	130	13.7	744	78.4	51	5.4		
Vocational school	246	18.8	1	0.4	105	42.7	122	49.6	18	7.3		
University	18	1.4	1	5.6	7	38.9	6	33.3	4	22.2		
**Occupation**												
Unemployed	111	8.5	5	4.5	54	48.6	47	46.8	5	4.5	251.56	<0.001*
Student	1,048	80.0	19	1.8	140	13.4	826	84.8	63	6.0		
Farmer	38	2.9	2	5.3	24	63.2	6	31.6	6	15.8		
Labor	113	8.7	5	4.4	66	58.4	33	37.2	9	8.0		
**Having regular income**												
No	1,180	90.1	24	2.0	213	18.1	864	73.2	79	6.7	102.35	<0.001*
Yes	130	9.9	7	5.4	71	54.6	48	36.9	4	3.1		
**Work experience outside village**												
No	1,099	83.9	20	1.8	203	18.5	108	51.2	11	5.2	53.78	<0.001*
Yes	211	16.1	11	5.2	81	38.4	804	73.2	72	6.6		
**Work experience abroad**												
No	1,294	98.8	29	2.2	273	21.1	910	70.3	82	6.3	26.88	<0.001 [a]
Yes	16	1.2	2	12.5	11	68.8	2	12.5	1	6.3		

* Significant level at α =0.05, [a] Fisher's exact test

Table 2 Substances use behaviors, knowledge of safe sex, and attitudes towards early sexual initiation behaviors and safe sex

**Characteristics**	**Total**		**Early sexual initiation**				**Never had sexual experience**		**No answer**		χ^2	**p-value**
			Yes (≤14)		No (>15)							
	n	%	n	%	n	%	n	%	n	%		
**Smoking**												
No	1,123	85.7	15	1.3	212	18.9	825	73.5	71	6.3	80.50	<0.001*
Yes	187	14.3	16	8.6	72	38.5	87	46.5	12	6.4		
**Alcohol use**												
No	1,019	77.8	13	1.3	162	15.9	777	76.3	67	6.6	123.18	<0.001*
Yes	291	22.2	18	6.2	122	41.9	135	46.4	16	5.5		
**Methamphetamine use**												
No	1,276	97.4	26	2.0	267	20.9	901	70.6	82	6.4	30.63	<0.001[a]
Yes	34	2.6	5	14.7	17	50.0	11	32.4	1	2.9		
**Heroin use**												
No	1,287	98.2	28	2.2	278	21.6	899	69.9	82	6.4	7.99	<0.036[a]
Yes	23	1.8	3	13.0	6	26.1	13	56.5	1	4.3		
**Crystal methamphetamine use**												
No	1,290	98.5	29	2.2	278	21.6	901	69.8	82	6.4	5.62	<0.088[a]
Yes	20	1.5	2	10.0	6	30.0	11	55.0	1	5.0		
**Level of knowledge of safe sex**												
Poor	1,045	79.8	25	2.4	191	18.3	758	72.5	71	6.8	37.01	<0.001[a]
Moderate	192	14.6	5	2.6	74	38.5	105	54.7	8	4.2		
Good	73	5.6	1	1.4	19	26.0	49	67.1	4	5.5		
**Level of attitudes towards early sexual initiation behaviors and safe sex**												
Poor	439	33.5	13	3.0	95	21.6	331	75.4	41	9.3	13.47	<0.036*
Moderate	574	43.8	13	2.3	118	20.6	443	77.2	26	4.5		
Good	297	22.7	5	1.7	71	23.9	221	74.4	16	5.4		

* Significant level at α =0.05, [a] Fisher's exact test

One-fourth of the participants used alcohol (22.2%), 14.3% smoked, and 2.6% used methamphetamine. A large proportion (79.8%) had a poor level of knowledge of safe sex, and 33.5% had a poor level of attitudes towards early sexual initiation behaviors and safe sex (Table 2).

Several substance use behaviors, knowledge of safe sex, and attitudes towards early sexual initiation behaviors and safe sex were found statistically significant among those who had –early sexual initiation, non-early sexual initiation, never had sexual experience, and non-response (Table 2).

Table 3 Comparisons of sexual behavior between sexes

Characteristics	Total n (%)	Male n (%)	Female n (%)	χ^2	p-value
**Total**	**1,310**	**514**	**796**	**N/A**	**N/A**
**Having boyfriend or girlfriend**					
No	806 (61.5)	315 (61.3)	491 (61.7)	0.21	0.885
Yes	504 (38.5)	199 (38.7)	305 (38.3)		
**Number of boyfriends or girlfriends**					
One	484 (94.0)	184 (92.4)	300 (98.4)	10.99	0.001*
More than one	30 (6.0)	15 (7.6)	5 (1.6)		
**Having sexual experience**					
No	912 (69.6)	323 (62.8)	589 (74.0)	19.01	<0.001*
Yes	315 (24.1)	148 (47.0)	167 (53.0)		
No answer	83 (6.3)	43 (51.8)	40 (48.2)		
**Age at first sexual initiation** (years)					
≤14	31 (13.6)	27 (87.1)	4 (12.9)	24.59	<0.001*
≥ 15	284 (86.4)	121 (42.6)	163 (57.4)		
Mean overall = 16.7 (SD=2.0); Median= 16, Mean in males = 16.0 (SD=1.92); Mean in females = 17.4 (SD=1.90)					
**Having sexual experience with one nightstand**					
No	231(73.3)	94 (40.7)	137 (59.3)	13.76	<0.001*
Yes	84 (26.7)	54 (64.3)	30 (35.7)		
**Having sexual experience with sex worker one year prior**					
No	291(92.4)	131 (45.0)	160 (55.0)	5.93	0.015*
Yes	24 (7.6)	17 (70.8)	7 (29.2)		
**Having regular partner**					
No	191 (63.6)	107 (56.0)	84 (44.0)	15.9	<0.001*
Yes	124 (36.4)	41 (33.1)	83 (66.9)		
**Alcohol or drug use prior having sexual initiation**					
No	284 (76.4)	130 (45.8)	154 (54.2)	1.80	0.406
Sometime	18 (4.5)	10 (55.6)	8 (44.4)		
Yes	13 (19.1)	8 (61.5)	5 (38.5)		

* Significant level at α =0.05

Five hundred and four (38.5%) participants had boyfriends or girlfriends, and among them, 6.0 % had more than one boyfriend or girlfriend. One-fourth (24.1%) of the participants had experienced sexual initiation (28.8% of males and 20.9% of females). Among those who had sexual initiation, 13.6% had sexual initiation before 15 years of age. The overall age at first sexual initiation was 16.7 years. After further examination, it was found that the average age at first sexual initiation among males was 16.0 years, with the minimum age at first sexual initiation being 12 years, whereas the average age at first sexual initiation among females was 17.4 years, with the minimum age being 13 years (Table 3).

Eighty-four participants (26.7%) had experienced having sexual initiation with one-night stands (which is having sexual experience without knowing their partner), 7.6% had ~~sexual experience with~~a sexual experience with a sex worker one year prior, and 19.1% regularly used alcohol or drugs prior to having sexual intercourse (Table 3).

In the comparisons of sexual behaviors between males and females, six factors were found to be statistical differences; number of boyfriends or girlfriends (p-value=0.001), having sexual experience (p-value<0.001), age at first sexual initiation (p-value<0.001), having sexual experience with one nightstand (p-value<0.001), having sexual experience with sex worker one year prior (p-value=0.050), and having regular partner (p-value<0.001) (Table 3).

Table 4 Univariable and multivariable analyses of factors associated with early sexual initiation among participants

Factors	Early sexual initiation		OR	95% CI	p-value	AOR	95% CI	p-value
	**Yes n (%)**	**No n (%)**						
**Total**	**31(9.8)**	**284 (90.2)**	**N/A**	**N/A**	**N/A**	**N/A**	**N/A**	**N/A**
**Sex**								
Male	27 (18.2)	121 (81.8)	9.09	3.10-26.67	<0.001*	7.30	2.06-25.83	0.002*
Female	4 (2.4)	163 (97.6)	1.00			1.00		
**Age** (years)								
15-17	19 (21.6)	69 (78.4)	6.54	2.13-20.08	0.001*	8.31	1.68-41.07	0.009*
18-20	8 (6.2)	120 (93.8)	4.13	1.71-9.93	0.002*	2.33	0.47-11.41	0.270
21-24	4 (4.0)	95 (96.0)	1.00			1.00		
**Tribe**								
Akha	12 (13.8)	75 (88.5)	1.00			1.00		
Lahu	4 (5.8)	65 (94.2)	0.38	0.11-1.25	0.112	0.57	0.11-2.78	0.493
Hmong	5 (9.4)	48 (90.6)	0.65	0.21-1.96	0.446	1.26	0.26-6.15	0.769
Yao	4 (16.0)	21 (84.0)	1.19	0.34-4.07	0.781	1.31	0.23-7.34	0.758
Karen	4 (8.9)	41 (91.1)	0.61	0.18-2.01	0.417	0.96	0.22-4.20	0.965
Lisu	2 (5.6)	34 (94.4)	0.36	0.07-1.73	0.206	0.40	0.05-3.08	0.380
**Religion**								
Buddhism	16 (8.6)	169 (91.4)	1.00			1.00		
Christianity	15 (11.5)	115 (88.5)	1.37	0.65-2.89	0.398	1.01	0.30-3.24	0.999

**Education**								
No education	2 (10.5)	17 (89.5)	1.00			1.00		
Primary school	3 (10.7)	25 (89.3)	1.02	0.15-6.76	0.984	1.77	0.12-25.24	0.673
≥High school	26 (9.7)	242 (90.3)	0.91	0.20-4.17	0.907	0.81	0.07-9.38	0.869
**Occupation**								
Student	19 (11.9)	140 (88.1)	1.00			1.00		
Unemployed	5 (8.5)	54 (91.5)	0.68	0.24-1.91	0.469	0.36	0.07-1.85	0.225
Farmer	2 (7.7)	24 (92.3)	0.61	0.13-2.80	0.529	0.55	0.05-5.82	0.625
Labor	5 (7.0)	66 (93.0)	0.55	0.20-1.56	0.266	0.38	0.06-2.25	0.290
**Having regular income**								
No	24 (10.1)	213 (89.9)	1.00			1.00		
Yes	7 (9.0)	71 (91.0)	1.14	0.47-2.76	0.767	1.41	0.29-6.79	0.669
**Smoking**								
No	15 (6.6)	212 (93.4)	1.00			1.00		
Yes	16 (18.2)	72 (81.8)	3.14	1.47-6.67	<0.003*	1.94	0.55-6.79	0.295
**Alcohol use**								
No	13 (7.4)	162 (92.6)	1.00			1.00		
Yes	18 (12.9)	122 (87.1)	1.83	0.86-3.89	0.112	0.48	0.15-1.55	0.222
**Methamphetamine use**								
No	26 (8.9)	267 (91.1)	1.00			1.00		
Yes	5 (22.7)	17 (77.3)	3.02	1.03-8.85	0.044*	1.83	0.30-11.05	0.508
**Heroin use**								
No	28 (9.2)	278 (90.8)	1.00			1.00		

Yes	3 (33.3)	6 (66.7)	4.96	1.17-20.93	0.029*	15.40	0.11-2044.62	0.273
**Crystal methamphetamine use**								
No	29 (9.4)	278 (90.6)	1.00			1.00		
Yes	2 (25.0)	6 (75.0)	3.19	0.61-16.56	0.166	0.12	0.01-22.58	0.431
**Work experience outside village**								
No	20 (9.0)	203 (91.0)	1.00			1.00		
Yes	11 (12.0)	81 (88.0)	1.37	0.63-3.00	0.420	2.21	0.73-6.66	0.156
**Work experience abroad**								
No	29 (13.4)	273 (90.4)	1.00			1.00		
Yes	2 (15.4)	11 (84.6)	1.71	0.36-8.10	0.498	4.03	0.42-38.18	0.224
**Level of knowledge of safe sex**								
Poor	25 (11.6)	191 (88.4)	2.48	0.31-19.38	0.385	1.90	0.11-32.08	0.656
Moderate	5 (6.3)	74 (93.7)	1.28	0.14-11.64	0.824	1.28	0.06-25.32	0.869
Good	1 (5.0)	19 (95.0)	1.00			1.00		
**Level of attitudes towards early sexual initiation behaviors and safe sex**								
Poor	13 (12.0)	95 (88.6)	1.94	0.66-5.70	0.226	0.76	0.18-3.17	0.715
Moderate	13 (9.9)	118 (90.1)	1.56	0.53-4.57	0.414	1.10	0.28-4.32	0.883
Good	5 (6.6)	71 (93.4)	1.00			1.00		

* Significance level at α =0.05

In the univariate model, five variables were found to be associated with early sexual initiation: sex, age, smoking, methamphetamine use, and heroin use. Males were 9.09 times (95%CI=3.10-26.67) more likely to have experienced early sexual initiation than females. Those aged 15–17 and 18–20 years were 6.54 times (95% CI = 2.13–20.08), and 4.13 times (95% CI = 1.71–9.93) more likely to have early sexual initiation than those aged 21–24 years, respectively. Those who smoked were 3.14 times (95% CI=1.47-6.67) more likely to have early sexual initiation than non-smoked. Those who used methamphetamine were 3.02 times (95% CI=1.03-8.85) more likely to have early sexual initiation than those who did not. Those who used heroin were 4.96 times (95% CI=1.17-20.93) more likely to have early sexual initiation than those who did not (Table 4).

In the multivariate model, sex and age were found to be associated with early sexual initiation. Males were 7.30 times (95%CI=2.06-25.83) more likely to have experienced early sexual initiation than females. Those aged 15–17 years were 8.31 (95% CI = 1.68–41.07) more likely to have early sexual initiation than those aged 21–24 years, respectively (Table 4).

Discussion

The hill tribe youths in Thailand used different substances, such as alcohol, tobacco, and amphetamines, and some of them used substances before having sexual initiation. One-fourth (24.0%) had sexual experience, while 6.3% did not respond whether they had experience or not. Among those who had sexual experience, 9.8% had their first sexual experience before the age of 15 years, and 87.1% were males. Being fFemales, and younger age were detected as the key contributors to having early sexual initiation among the hill tribe youths in Thailand.

In our study, it was found that males (12 years old) had an earlier age of having first sexual initiation than females (13 years old). However, the overall age at first experiencing in sexual initiation among the hill tribe youths was 16.7 years: 16.0 years old among males,sexual initiation among the hill tribe youths was 16.7 years: 16.0 years old among males and 17.4 years old among females. In a study in South Africa among school children between grades 9 and 11 [30], it was reported that 50.4% of males and 30.1% of females had sexual experience. The median age at first sexual initiation for males and females was 16.5 years and 17.5 years, respectively. A study of Portuguese female students reported that the mean age of their first sexual initiation was 16.4 years old, which was younger than that of hill tribe women (16.7 years old); however, fewer people in the Portuguese study used substances (9.0%) before engaging in sexual initiation than did the hill tribe youths in Thailand (21.3%). A study in Ethiopia [31] reported that the mean age for sexual initiation among youths who had

experienced sexual initiation was 15.4 years, while the corresponding age was 16.2 years among the hill tribe youths in Thailand. A study in Ireland [32] reported that a greater proportion of girls (22.8%) than boys (13.4%) had sexual initiation prior to 14 years of age, in contrast to the findings for the hill tribe youths. A study conducted among the children who were attending school in the central region of Thailand [33] reported no sex-based differences in the proportion of early sexual experience among the youths (24.3% males and 24.0% females); however, 35.0% of the youths had more than one sexual partner. This rate is lower than that of the hill tribe youths having sexual initiation (30.4%), with a greater proportion of males than females having sexual initiation. In one more study [34] conducted among high school children aged 16-20 years living in rural northern areas of Thailand, it was found that 29.5% had experienced sexual initiation: 35% in males~~,~~ and 25.5% in females. Tangmunkongvorakul~~, et al. [34] also reported that the average age of having first sexual initiation was 15.8 years: 15.9 years in males,~~ et al. [34] also reported that the average age of having first sexual initiation was 15.8 years: 15.9 years in males and 15.8 years in females. The hill tribe youths have a younger age of their first sexual initiation than Thai youths, which might be the culture of the hill tribe that needs to get married early to have a child for help in their family work. This is clearly supported by a study by Detpitukyon~~, et al. stated~~ et al. stating that Lahu people need to ~~get married in early life~~marry early to support their family [35].

Early sexual behavior among the hill tribe was found in a larger proportion of males than females. This coincides with a longitudinal analysis conducted among Latin American youths, which reported that males had greater odds of having early sexual initiation than females [36]. Gazendam, et al. [37] also reported that being boys was a greater chance to have early sexual initiation than girls in Canada. A study in Nigeria [38] supported that being male was detected as the factor associated with having early sexual initiation among high school students. A study among vocational students in northern Thailand reported that males were detected as one of the factors associated with having early sexual initiation, particularly those who used alcohol [39]. The male gender dominant [35] and the culture of having early marriage among the hill tribe could lead them to have early sexual initiation.

Our study found that the younger age group of the hill tribe youths have a greater chance to have early sexual initiation than those older youths significantly. ~~There are almost 10-year intervals between 15 and 24 years of the youths in our study~~Our study has almost 10-year intervals between 15 and 24 years of the youths, ~~.~~ ~~-~~t~~T~~hen with a wide interval range of age~~, younger ages might be exposed to new technology and mass media of pornographic materials and seeking partners~~; younger ages might be exposed to new technology and mass media of

pornographic materials and seeking partners, which leads them to have early sexual initiation. Lay~~, et al [40] reported that younger youths both boys and girls~~ et al. [40] reported that younger youths, both boys and girls, were associated with early sexual initiation among Brazilian adolescents.

Several substances use behaviors were associated with early sexual initiation among the hill tribe youths including smoking, methamphetamine use, and heroin use. Exposing Substances could lead to uncontrol individuals' emotions, particularly young age people, and having sexual intercourse eventually. A systematic review of cohort studies reported that youths' substance use behaviors were one of the strong predictors of early sexual initiation [41]. A study conducted in the United States also reported that substance use increased early sexual initiation [42]. Substance use was a significant predictor of early sexual initiation among young people in Brazil [43] and Kenya [44].

In our study, it was found that 83 participants (6.3%) did not respond to the question asking about sexual initiation experience. Tamorpark~~, et al [45] reported that asking a sensitive question particularly sexual behaviors and substance use by audio computer assisted self-interview~~ et al. [45] reported that asking a sensitive question, particularly sexual behaviors and substance use, by audio computer-assisted self-interview gained a higher accuracy than a paper-based questionnaire. With the circumstance of the hill tribe culture that they are not familiar with discussing sexual behaviors with anyone, then this is not an overestimate for the researchers. In several traditional cultures, especially marriage in early life [35]~~, and parenting styles in keeping sexual expression uncommon discussion among family members [20], young people tend to learn sexual education from~~ and parenting styles in keeping sexual expression uncommon discussion among family members [20], young people tend to learn sexual education from the media. Moreover, several hill tribe youths move to the city for education or work, the opportunity to be exposed to early sexual initiation at an early age. The major driving factors that ~~to contribute~~contribute to the hill tribe youths' exposure to early sexual initiation are poverty and limited literacy ~~poor education~~ in both their parents and themselves.

A few limitations were found during the study. Many questions on the study protocol inquire about sexual behaviors, while some tribes, such as Lahu women, do not commonly talk about sexual behavior in their culture, particularly in public areas. Therefore, the findings on sexual behaviors could be underestimated in some aspects; however, the researchers explained to the participants as thoroughly as possible that their information would remain private and confidential and that it could not be used to identify the participants later on. During the process of data collection, all essential information, including concerns regarding privacy and

confidentiality, was carefully explained to all participants to increase their willingness to answer the questions truthfully. The participants cooperated well with this aspect of the study. One more point, the number of participants in each tribe was different which could lead to the quality of the generalization of the findings to the specific tribe. Finally, a few participants did not clearly understand the context of some questions due to limited literacypoor education. To address this issue, the researchers personally explained the meaning and content of confusing items. While the study showed various strengths such as the concept of investigating the very sensitive issue on investigating early sexual initiation among the marginalized population among six main hill tribes,The study showed various strengths, such as the concept of investigating the very sensitive issue of early sexual initiation among the marginalized population among six main hill tribes and the large number of participants recruited into the study.

Conclusion

Hill tribe youths are facing a problem of early sexual initiation, particularly male youths. Several substances are contributing to early sexual initiation among the hill tribe youths. Therefore, the interventions to reduce early sexual initiation behavior among the hill tribe youths should be focused on male gender and substance use. Understanding the context social norms and culture of sexual activity and the ability to discuss the issue at the family level are also critical points. The intervention of sexual education among family members, community-oriented, and extracurricular to add to the formal education in Thailand should be implemented.

Supplementary information

S1 file 1. Questionnaire used in the study.

S2 file 2. Study data

Acknowledgements

We would like to thank all village headmen for their kind support in accessing the participants. We also would like to thank all participants for providing essential information in the study.

Abbreviations

CI	Confident interval
HIV	Human immunodeficiency virus
IOC	Item-objective congruence
UN	United Nations

WHO World Health Organization

Declarations

Author Contributions

Conceptualization: Tawatchai Apidechkul, Ratipark Tamornpark

Data curation: Tawatchai Apidechkul, Ratipark Tamornpark, Chalitar Chomchoei, Panupong Upala, Fartima Yeemard.

Formal analysis: Tawatchai Apidechkul, Ratipark Tamornpark

Funding acquisition: Tawatchai Apidechkul.

Investigation: Tawatchai Apidechkul, Ratipark Tamornpark, Chalitar Chomchoei, Panupong Upala, Fartima Yeemard.

Methodology: Tawatchai Apidechkul, Ratipark Tamornpark

Project administration: Tawatchai Apidechkul.

Supervision: Tawatchai Apidechkul, Ratipark Tamornpark

Writing – original draft: Tawatchai Apidechkul, Ratipark Tamornpark, Chalitar Chomchoei, Panupong Upala, Fartima Yeemard.

Writing – review & editing: Tawatchai Apidechkul, Ratipark Tamornpark, Chalitar Chomchoei, Panupong Upala, Fartima Yeemard.

References

1. United Nations (UN). World youth report 2018: youth and the 2030 agenda for sustainable development, UN, New York. 2018. doi: https://doi.org/10.18356/0c6f53e0-en.

2. Anguzu G, Flynn A, Musaazi J, Kasirye R, Atuhaire LK, Kiragga AN, et al. Relationship between socioeconomic status and risk of sexually transmitted infections in Uganda: multilevel analysis of a nationally representative survey. Int J STD AIDS. 2019; 30(3): 284-291. DOI: https://doi.org/10.1177/0956462418804115. Epub 2018 Nov 11.

3. World Health Organization (WHO). Responding to children and adolescents who have been sexually abused: WHO clinical guidelines. Geneva: World Health Organization; 2017. Available from: https://apps.who.int/iris/bitstream/handle/10665/259270/9789241550147-eng.pdf;jsessionid=C5DCAF1BDBE1C5E0A3AD4F7F8331EC12?sequence=1

4. Wesche R, Kreager DA, Feinberg ME. Peer acceptance and sexual behaviors from adolescence to young adulthood. J Youth Adolescence. 2019; 48: 996. 1-20. DOI: https://doi.org/10.1007/s10964-019-00991-7

5. Epstein M, Manhart LE, Hill KG, Bailey JA, Hawkins JD, Haggerty KP, et al. Understanding the link between early sexual initiation and later sexually transmitted

infection: test and replication in two longitudinal studies. J Adolesc Health. 2014; 54(4): 435-441

6. Speizer I, Okigbo CC. Determinants of sexual activities and pregnancy among unmarried young women in urban Kenya: a cross-sectional study. PLoS One. 2015; 10(6): e0129286.

7. Glynn JR, Sunny BS, DeStavola B, Dube A, Chihana M, Price AJ. Early school failure predicts teenage pregnancy and married: a large population-based cohort study in northern Malawi. PLos One. 2018; 13(5): e0196041.

8. Yaya S, Bishwajit G. Age at first sexual intercourse and multiple sexual partnerships among women in Nigeria: a cross-sectional analysis. Front Med (Lausanne). 2018; 5:171. DOI: https://doi.org/10.3389/fmed.2018.00171.

9. Brown JL, Gause NK, Northern N. The Association between alcohol and sexual risk behaviors among college students: a review. Curr Addict Rep. 2016; 3(4): 349-355. DOI: https://doi.org/10.1007/s40429-016-0125-8.

10. Yazdi V, Mehrolhassani M, Zolala F, Haghdoost A, Oroomiei N. Determinants of risky sexual practice, drug abuse and alcohol consumption in adolescents in Iran: a systematic literature review. BMC Reprod Health. 2019; 16: 115-125.

11. Landry M, Turner M, Vyas A, Wood S. Social media and sexual behavior among adolescents: Is there a link? JMIR Public Health Surveill 2017;3(2). DOI: https://doi.org/10.2196/publichealth.7149.

12. Merrilla RA, Liang X. Associations between adolescent media use, mental health, and risky sexual behaviors. Child. Youth Serv Rev 2019; 103: 1-9. DOI: https://doi.org/10.1016/j.childyouth.2019.05.022

13. World Health Organization (WHO). High rates of unintended pregnancies linked to gaps of family planning. Available from: https://www.who.int/news-room/detail/25-10-2019-high-rates-of-unintended-pregnancies-linked-to-gaps-in-family-planning-services-new-who-study.

14. Thutsanti P, Apidechkul T, Inta C, Upala P, Tamornpark R. Adaptation to Thai citizenship: a case study of Thai-Chinese Yunnan, Mae Fah Laung District, Chiang Rai, Thailand. J Health Sci Altern Med. 2019; 1(1): 8-13.

15. Chomchoei C, Apidechkul T, Wongnuch P, Tamornpark R, Upala P, Nongkhai MPN. Perceived factors influencing the initiation of methamphetamine use among Akha and Lahu youths: a qualitative approach. BMC Public Health. 2019;19(847): 1-11. DOI: https://doi.org/10.1186/s12889-019-7226-y.

16. Princess Maha Chakri Siridhorn Anthropology Center. Hill tribe. Available from: http:// www.sac.or.th/main/index.php. Assessed 15 Nov 2019

17. Department of Mineral Resources. Geology of Thailand. Ministry of Natural Resources and Environment. 2016. Available from: http://www.dmr.go.th/main.php?filename=GeoThai_En
18. Apidechkul T, Wongnuch P, Sittisarn S and Ruanjai T. Health situation of Akha hill tribe in Chiang Rai Province, Thailand. J Pub Health Dev. 2016; 14(1):77-97.
19. Jakae O, Tanpichai P, Jaiaree A. Construction of social space in Thai hill tribe ethnic groups in Kamphaeng Phet province. Kasetsart Journal of Social Sciences. 2018. 1-7. DOI: https://doi.org/10.1016/j.kjss.2018.07.019
20. Singkorn O, Apidechkul T, Putsa B, Detpetukyon S, Sunsern R, Thutsanti P, et al. Factor associated with alcohol use among Lahu and Akha hill tribe youths, northern Thailand. Subst Abuse Treat Prev Policy. 2019; 14(5); 1-14. doi: 10.1186/s13011-019-0193-6.
21. Aurpibul L, Tangmunkongvorakul A, Musumari PM, Srithanaviboonchai K, Tarnkehard S. Patterns of sexual behavior in lowland Thai youth and ethnic minorities attending high school in rural Chiang Mai, Thailand. PLoS One. 2016; 11(12). 1-17. DOI: 10.1371/journal.pone.0165866.
22.Thomas PA, Liu H, Umberson D. Family relationships and well-being. Innov Aging. 2017; 1(3).1-11. DOI: 10.1093/geroni/igx025.
23. Apidechkul T. Sexual behaviors and seroprevalence of HIV, HBV, and HCV among hill tribe youths of Northern Thailand. BMC Public Health. 2019; 19(1101). 1-17. DOI: https://doi.org/10.1186/s12889-019-7459-9
24. Chunuan S, Kosunvanna S, Sripotchanart W, Lawantrakul J, Lawantrakul J, Pattrapakdikul U, et al. Characteristics of abortion in southern Thailand. Pacific Rim Int J Nurse Res. 2012; 16(2): 97-112.
25. Apidechkul T, Laingoen O, Suwannaporn S. Inequity in accessing health care service in Thailand in 2015: a case study of the hill tribe people in Mae Fah Luang District, Chiang Rai, Thailand. J Health Res. 2016; 30 (1): 67-71.
26. Apidechkul T. Epidemiology of the hill tribe HIV/AIDS populations, Thailand. J Med Assoc Thai. 2016; 99(6): 702-710.
27. The Hill tribe Welfare and Development Center, Chiang Rai Province. Hill tribe population. In: The hill tribe welfare and development center. Chiang Rai: Ministry of Social Development and Human Security; 2018. p. 17–25.
28. Pouhoseingholi MA, Vahedi M, Rahimzadeh. Sample size calculation in medical studies. Gastroenterol Hepatol Bed Bench. 2013; 6(1): 14-17.

29. Franca MT, Frio GS. Factors associated with family, school and behavioral characteristics on sexual initiation: a gender analysis for Brazilian adolescents. PlosOne. 2018; 13(12). 1-16. DOI: 10.1371/journal.pone.0208542

30. Amoateng AY, Kaule-Sabiti I. Biosocial correlates of age at first sexual intercourse: the case of grade 9 and grade 11 pupils in the northwest province of South Africa. Journal of Biosocial Science. 2014; 1-17. DOI: 10.1017/S0021932014000492.

31. Girmay A, Mariye T. Risky sexual behavior practice and associated factors among secondary and preparatory school students of Aksum town, northern Ethiopia, 2018. BMC Res Nores. 2019; 12(698): 1-7. DOI: 10.1186/s13104-019-4714-1.

32. Young H, Burk L, Gabhainn SN. Sexual intercourse, age of initiation and contraception among adolescents in Ireland: findings from the health behavior in school-aged children (HBSC) Ireland. BMC Public Health. 2018; 18(362). 1-17. DOI: 10.1186/s12889-018-5217-z.

33. Srijaiwong S, Sindhu S, Ratinthorn A, Viwatwongkasem C. Factors influencing sexual behaviors among Thai adolescents. Journal of Population and Social Studies. 2017; 25(3): 171-193.

34. Tangmunkongvorakul A, Srithanaviboonchai K, Guptarak M, Wichajarn M, Yungyuankul S, Khampan R, et al. Attitudes and behavior among rural Thai adolescents regarding sexual intercourse. Southeast Asian J Trop Med Public Health. 2014; 45(6): 1437-1447.

35. Detpitukyon S, Apidechkul T, Sunsern R, Anuwatnonthakate A, Singhhorn O, Putsa B, et al. Pattern and perception of alcohol drinking among the Lahu people, northern Thailand. Journal of Health Research. 2018; 32(3): 185-197.

36. Cueto S, Leon J. early sexual initiation among adolescents: a longitudinal analysis for 15-year-olds in Peru. Interamerican Journal of Psychology. 2016; 50(2): 186-203.

37. Gazendam N, Cleverley K, King N, Pickett W, Phillip SP. Individual and social determinants of early sexual activity: a study of gender-based differences using the 2018 Canadian Health Behavior in school-aged children study (HBSC). PlosOne. 2020; 15(9): 1-13. DOI: 10.1371/journal.pone.0238515.

38. Durowade KA, Babatunde OA, Omokanye LO, Elegbede OE, Ayodele LM, Adewoye KR, et al. Early sexual debut: prevalence and risk factors among secondary school students in Ido-ekit, Ekiti state, South-West Nigeria. Afr Health Sci. 2017; 17(3): 614-622.

39. Liu A, Kilmarx P, Jenkins RA, Manopaiboon C, Mock PA, Jeeyapunt S, et al. Sexual initiation, substance use, and sexual behavior and knowledge among vocational students in northern Thailand. International Family Planning Perspective. 2006; 32(3): 126-135.
40. Lay AA, Fujimori E, Duarte LS, Borges AL. Prevalence and correlates of early sexual initiation among Brazilian adolescents. Plosone. 2021; 16(12).: e0260815.
41. Reis LF, Surkan PJ, Atkins K, Garcia-Cerde R, Sanchez ZM. Risk factors for early sexual intercourse in adolescence: a systematic review of cohort studies. Child Psychiatry and Human Development. 2023. DOI:10.1007/s10578-023-01519-8.
42. Clark DA, Donnellan MB, Durbin CE, Nuttall AK, Hicks BM, Robins RW. Sex, drugs, and early emerging risk: examining the association between sexual debut and substance use across adolescence. Ploseone. 2020;15(2): e0228432.
43. Reis LF, Surkan PJ, Valente JY, Bertolla MH, Sanchez ZM. Factors associated with early sexual initiation and unsafe sex in adolescents: substance use and parenting style. Journal of Adolescence.2020; 79: 128-35.
44. Sing'oei V, Owuoth JK, Otieno J, Yates A, Aadalagu B, Smith HJ, et al. early sexual debut is associated with drug and decreased educational attainment among males and females in Kisumu country, Kenya. Reproductive Health. 2023; 20(11). DOI: 10.1186/s12978-023-01639-3.
45. Tamornpark R, Mulikaburt T, Adplong A, Upala P, Chomchoie C, Yeemard F. Effectiveness of the hill tribe audio computer assisted self interview versus self administration questionnaire to assess sexual behaviors among the hill tribe youths. 2021;3(3): 22-29

Response to the reviewer comment

PONE-D-23-33898R1
Influencing factors early sexual initiation among hill tribe youths in Chiang Rai Province, northern Thailand: A community-based cross-sectional study
PLOS ONE

Dear Editor and Reviewers,

Thank you very much for the opportunity to revise that paper. We have carefully and completely revised your comments. The paper also double-checked the grammatical error before re-submitting. Please kindly the following our responses.

Thank you,

TK

+++

Please use the space provided to explain your answers to the questions above. You may also include additional comments for the author, including concerns about dual publication, research ethics, or publication ethics. (Please upload your review as an attachment if it exceeds 20,000 characters)

Reviewer #2: Reviewers' comments
Title: Influencing factors early sexual initiation among hill tribe youths in Chiang Rai Province, northern Thailand: A community-based cross-sectional study
The manuscript was well-prepared. I have a few comments as follows.
Abstract
-The conclusion did not come from the study findings. The authors can be more specific, like suggesting what to be include in the training program (what knowledge is lacking from the results).
Methods

: It is a sexual education focused on safe sex and early sexual interaction protection.

-The authors mentioned that the questionnaires were piloted in Mae Chan district. Where did the study take place? Please add in the study setting.

: Thank you. It's added which are Huan Hin Fon and Sun Ti Suk Villages

-Please clarify about the recruitment which should happen without coercion. The authors stated that they contacted village headmen for their permission to access the villages, but how those youth was reached? As most of them were studying, were they

in the village or lived somewhere outside the village for their schooling? Could any youth deny participation? Did their parents, family members, and other villagers know about their entering the study? How many of them refused to join? Any compensation to youth, their family, or villager headmen?

: There was nobody to refuse the participants. In the process of recruitment, after providing them with information about the study, all participants were informed that they had the right to refuse or deny joining the project as the standard protocol for human research ethical procedure. All parents were also informed about the study, and only those who had children aged less than 17 and below were asked for their permission to gather the information from the participants (youths). All participants and parents agreed and were willing to join the project. During the study, we did not have any inclusion and exclusion criteria for whether the participants were in or out of the school. All participants were compensated 200 baht ($6), which is clearly declared in the ethical consideration process.

-Please describe how youth were instructed before completing the questionnaires. Were there any specific guide written in the questionnaire or mentioned verbally, like the time frame for alcohol, smoking, and substance use (lifetime, or any specific duration to be reported), definition of sexual intercourse, etc.)

: Each question provided complete and clear information. We did not have any specific guide for completing the questionnaire; however, all participants were told that if there was any confusion, they could contact the researcher. Nobody asked to clear any questions.

: Since we worked a cross-sectional, then the questions were asked directly with the time frame, except for some question that we had a clear its frame in the questions.

Results

Table 1: The column “Early sexual initiation” can list only “yes” (and add the denominator somewhere). Similarly for variables with yes/no response, presentation in the table can be only yes which would make them easier to read and reduce the table size.

: Thank you for the comments; however, after discussing with the team, we decided to maintain this column and information to make it easier and quicker for the readers to go through the details.

Discussion

Page 18 line 17-18. The study found that a larger proportion of male had early sex initiation than female youth. Was it a result of reporting bias? Males perceived power

over females when they initiated sex, while females might have early sex without their willingness. Thus, males were more likely to report having early sex. It might be related to patriarchy in Thai society, which could be prominent in hill tribe cultures. Just a thought, I know the authors are experts on this issue. Could the authors provide any comments or discussion on this possibility.

: Thank you for this great idea. We had discussed this point while making the conclusion; however, with the study design, we could not extend our thoughts outside of what we found in the results. One of our team is trained in medical anthropology and is trying to work on her project to understand some critical issues from our findings, particularly gender roles and sexual inequality in some tribes. We very much hope that we will be able to publish our findings soon.

Page 19 line 32" the term "poor education" might not appropriate and too broad, given that most participants had attended school. The authors could consider changing to other words like "limited literacy", or "low reading ability".

: Thank you very much for the suggestion; we agree. It's changed it to limited literacy.

Thank you very much,

TK

Assoc Prof. Dr. Tawatchai Apidechkul, MSc (Infectious Epidemiology), Dr. P. H (Epidemiology)

Director, Center of Excellence of Hill Tribe Health Research,

School of Health Science, Mae Fah Luang University, Thailand

Former Hubert H Humphrey Fellow (2013-2014), Emory University
