# Supplementary material for: Factors influencing early sexual initiation among hill tribe youths in Chiang Rai Province, Northern Thailand: A community-based cross-sectional study
Source: PLoS One. 2025 Apr 8;20(4):e0321083. doi: 10.1371/journal.pone.0321083 (PMC11991288; doi:10.1371/journal.pone.0321083)
Supplement: Attachment — Submitted filename: Response to reviwers comments.pdf [file pone.0321083.s008.pdf]

Response to reviewer comments

Reviewer #2: Reviewers' comments
Thank you for the revised manuscript. The authors have done a great job in improving it.
I have only a few comments as follows.
Abstract
Page 2 line #3-4: males was compared to males (one should be females?)

: We apologize for this mistake; it should read female. Please see line 6, page 2.

Page 2 line# 12: impact of substance use was mentioned in the conclusion, but no information in the results of abstract to explain how they were related. I understand it later when reading results part in the main text. Could the authors add a sentence about substance use in the results to guide the readers that the conclusion did not come from nowhere?
Methods

: Thank you for this helpful comment. This occurs because the statistics are significant in the univariate analysis and chi-square test. However, in the multivariable analysis, there were no associations with early sex. Thus, we slightly extended our thoughts in the Conclusion sections. We have also added a sentence to the Abstract (page 2, lines 2-3).

Page 4 line#15: "…, it needed 1293 samples in the study" What is it refer to? Should the author modify it to something like "we needed 1293 participants in the study"

: Thank you; it has been revised. Please see page 4, lines 12-13.

Page 4 line#29: "Those questions that scored less than 0.5 were excluded." I think it should be "less than or equal to 0.50", isn't it? Please recheck.

: Sorry for the mistake, it is 0.5. We have revised the sentence in the text as follows: "Those questions that scored less than 0.5 were excluded, questions that scored 0.50-0.69 were revised as comments…"

Results
Page 7 line # 14: It seemed like the word "difference" is missing from the 3rd paragraph. What was statistically significant is the difference, isn't it? Please check.

: Thank you; this information was added. Please see page 7, line 14.

Page 13 line#3-4.: the percentage was different from other place (13.6% had sexual initiation before 15 years of age", it was 9.8% elsewhere). Please recheck.

: Thank you so much. This small error prompted us to check the entire paper. Now, all the points are correct.

Page 13 line#13-17: P-value in the text should be written only "p", usually small p with italic. Discussion

Thank you for the comment. We have discussed this point, and we have to maintain its original form because two of our statisticians inform us that there is a specific requirement for the journal. However, using the full term is also common, as used in our previous papers. We hope that you understand.

Page 17 line#21: I think it should be "Males and younger age…" not females.

: Thank you so much for calling this to our attention; we have corrected it accordingly.

Page 18 line # 31-33: The sentence is confusing, please revise the language.

: Thank you for the comment. These sentences were drafted by one of our coauthors during the manuscript development. However, we forgot to include the details of the reference. Once we read the whole paragraph, it still clearly presents the information we want to present. However, we deleted the sentences from the text. Thank you so much for pointing this out.

Page 19 line# 9: the phrase ", it was found that" could be removed as it has no meaning in the sentence.

: Thank you; this has been deleted.

Page 19 line#17-18: Please add numbers from the study finding to support the statement as the authors did in other statements.

: Thank you for this helpful comment. We have added this number to the paragraph. Please see page 19, lines 16-17.

Language quality
Overall, the language in this article is difficult to read, not flow while reading. I would advise the article to be edited by native English speaker for quality improvement.

: Thank you; the paper has been double-checked by AJE (certificate no: 2012-7831-AE6C-33FF-026E)

Reviewer #3: I can see the authors have improved previous versions of the manuscript, based on reviewers' comments. The study is of importance because there are important results that should be known in the country and other countries with similar situations. However, despite improvements, there are several places where the descriptions and language used need to be revised in order to make it clear for the reader. I have made a number of suggestions to edit the document in the attached file. Please after carrying out corrections have the document checked by someone who speaks and writes English, so it is fully edited. Thanks.

: Thank you. The paper has been double-checked by AJE (certificate no: 2012-7831-AE6C-33FF-026E.).

1. Check the number/information in percentage of having had sexual experience.

The text has been checked and corrected; see page 1, lines 33-35.

2. Page 2, lines 27-28, delete “old; these behaviors vary in different societies frequently occur among youths [13]”

: This has been deleted per your suggestion.

3. Page 3, line 1, “lived”… “live”

: This has been deleted per your suggestion.

4. Page 3, lines 10, delete “the hill tribe youths who have been clearly defined as”

: This has been deleted per your suggestion.

5. Page 3, delete “then” in line18

6. Many points in the whole paper, in the PDF file, are asked to improved.

: All the points have been improved by AJE.

TK

Assoc Prof. Dr. Tawatchai Apidechkul, MSc (Infectious Epidemiology), Dr. P. H (Epidemiology)

Director, Center of Excellence of Hill Tribe Health Research,

School of Health Science, Mae Fah Luang University, Thailand

Former Hubert H Humphrey Fellow (2013-2014), Emory University
