# Supplementary material for: Factors influencing early sexual initiation among hill tribe youths in Chiang Rai Province, Northern Thailand: A community-based cross-sectional study
Source: PLoS One. 2025 Apr 8;20(4):e0321083. doi: 10.1371/journal.pone.0321083 (PMC11991288; doi:10.1371/journal.pone.0321083)
Supplement: Attachment — Submitted filename: Response to editor and reviewers_PONE-D-20-21332.docx [file pone.0321083.s003.docx]

**Response to editor and reviewers’ comments**

PONE-D-20-21332
Factors associated with early sexual intercourse among hill tribe youths in northern Thailand: A cross-sectional study
PLOS ONE

Dear editor,

Under this revised version, we have made impartments in all points raised by redrawers and editor’s comments. The abstract has been revised. The aim of the study and the surrounding information were improved. Information about the hill tribe including the sample size calculation in the study was cleared and presented as a generalization of the findings to the population. The method of sample selection and process of data gathering were improved. Variables in the study were defined properly as comments. We added tables 2 and 3 to present information on substance use and sexual behaviors including information on early sexual intercourse between males and females including the summaries. The whole parts of the discussion and conclusion were revised and improved as per your critical comments. Finally, all references have been carefully revised according to the journal guidelines.

We do hope that you are happy with this version.

Regards,

TK

Journal Requirements:

When submitting your revision, we need you to address these additional requirements.

1. Please ensure that your manuscript meets PLOS ONE's style requirements, including those for file naming. The PLOS ONE style templates can be found at

<https://journals.plos.org/plosone/s/file?id=wjVg/PLOSOne_formatting_sample_main_body.pdf> and

<https://journals.plos.org/plosone/s/file?id=ba62/PLOSOne_formatting_sample_title_authors_affiliations.pdf>

 : Thank you, we have checked and reformatted in whole text according to the journal style.

2. Please include additional information regarding the survey or questionnaire used in the study and ensure that you have provided sufficient details that others could replicate the analyses. For instance, if you developed a questionnaire as part of this study and it is not under a copyright more restrictive than CC-BY, please include a copy, in both the original language and English, as Supporting Information.

 :Thank you. We have attached both Thai and English versions of the questionnaires.

3. In your Methods section, please provide additional information about the participant recruitment method and the demographic details of your participants. Please ensure you have provided sufficient details to replicate the analyses such as: a) the recruitment date range (month and year),

: Data were collected between November 2019 and March 2020, page 5, lines 13.

b) a description of any inclusion/exclusion criteria that were applied to participant recruitment,

: It was made clearer on the points of inclusion and exclusion criteria in page 4, lines 3-6.

c) a table of relevant demographic details,

: I am not sure to put a table into the method section is suitable, then, I have added information of the hill tribe people in the study location in page 3, lines 2932-. We do hope you understand and we happy to add more information is required.

d) a statement as to whether your sample can be considered representative of a larger population

: Since 5 villages in each tribe were randomly selected from the whole lists of the hill tribe villages in Chiang Rai, and all youths lived in selected villages were invited into the study if they were met the inclusion and exclusion criteria.

: This is a community-based cross-sectional design, then to get the participants by selecting the villages from the list by random method. The invited targeted populations in villages are a good representative of the populations. If any point that we could not make an explanation, please feel free to suggest your comments on these points.

e) a description of how participants were recruited, and descriptions of where participants were recruited and where the research took place

: Thank you for the comment. Basically, we invited all youths aged 15-24 years who lived in the selected villages into the study on a voluntary basis after providing them with the study information in advance by a village headman. Please see the detail on page 5, lines 11-13.

f) Moreover, please clarify how variables included in the analysis were defined and categorized.

 : The categorical of variables are explained between page 5, lines 21-34, and page 6, lines 1-2.

4. Thank you for stating the following in the Acknowledgments Section of your manuscript:

"Finally, we also would like to thank The National Research Council of Thailand, Mae Fah
Luang University, and The Center of Excellence to the Hill tribe Health Research for support
grant."

We note that you have provided funding information that is not currently declared in your Funding Statement. However, funding information should not appear in the Acknowledgments section or other areas of your manuscript. We will only publish funding information present in the Funding Statement section of the online submission form.

Please remove any funding-related text from the manuscript and let us know how you would like to update your Funding Statement. Currently, your Funding Statement reads as follows:

"The study was funded by The National Research Council of Thailand and Mae Fah Laung University, Thailand (Grant No.06/2560). Dr. Tawatchai Apidechkil was granted. However, the funders have no role and involvement in the study. "

Please include your amended statements within your cover letter; we will change the online submission form on your behalf.

: Thank you for the comments.

: We have removed the statement of acknowledgment of the funders from the acknowledgment and put it into the finding section.

[Note: HTML markup is below. Please do not edit.]

Reviewers' comments:

Reviewer's Responses to Questions

**Comments to the Author**

1. Is the manuscript technically sound, and do the data support the conclusions?

The manuscript must describe a technically sound piece of scientific research with data that supports the conclusions. Experiments must have been conducted rigorously, with appropriate controls, replication, and sample sizes. The conclusions must be drawn appropriately based on the data presented.

Reviewer #1: Partly

Reviewer #2: Yes

2. Has the statistical analysis been performed appropriately and rigorously?

Reviewer #1: I Don't Know

Reviewer #2: Yes

3. Have the authors made all data underlying the findings in their manuscript fully available?

The [PLOS Data policy](http://www.plosone.org/static/policies.action#sharing) requires authors to make all data underlying the findings described in their manuscript fully available without restriction, with rare exception (please refer to the Data Availability Statement in the manuscript PDF file). The data should be provided as part of the manuscript or its supporting information, or deposited to a public repository. For example, in addition to summary statistics, the data points behind means, medians and variance measures should be available. If there are restrictions on publicly sharing data—e.g. participant privacy or use of data from a third party—those must be specified.

Reviewer #1: No

Reviewer #2: Yes

4. Is the manuscript presented in an intelligible fashion and written in standard English?

PLOS ONE does not copyedit accepted manuscripts, so the language in submitted articles must be clear, correct, and unambiguous. Any typographical or grammatical errors should be corrected at revision, so please note any specific errors here.

Reviewer #1: No

Reviewer #2: Yes

5. Review Comments to the Author

Please use the space provided to explain your answers to the questions above. You may also include additional comments for the author, including concerns about dual publication, research ethics, or publication ethics. (Please upload your review as an attachment if it exceeds 20,000 characters)

Reviewer #1:

1. The pages nor the lines have not been number, which makes difficult to make reference to specific sections of the manuscript during review

: Sorry for the difficulty, we have put lines on all pages.

2. The research problem has not been adequately articulated to provide enough warrant for this study among the Hill tribe. Indeed you suggest that the hill tribe have peculiar lifestyle patterns, beliefs, perceptions and acceptance of sexual intercourse, yet you fail to explain or describes any of such in relation to why this particular study is warranted.

: Thank you for the great comment. We have added information on page 3, lines 18-21 with references.

3. What are the pathways through which globalization increase the risk of Hill tribe youth engaging in unexpected sexual intercourse as you suggest?

: The public or social media including the migration to work in the cities among the hill tribe youths. Accessing social media, they consume various information and movies. While most of the hill tribe people have poor education, clarifying accurate and updated information is very difficult for them. Moreover, those working in big cities to get a better income to support their family both inside Thailand and abroad are able to exposed to substances that could lead to unexpected sexual intercourse. This is the real situation among the hill tribe's young generations in Thailand.

4. To title does not quite align with the aims of the study as indicated in the end of the Introduction section. The title focuses on early sexual intercourse while the aims (and indeed a large section of the results) talk about sexual behaviours in general. This makes it difficult to piece together the thrust of the manuscript

: Sorry for the point. We totally agree with you. Basically, we intended to assess factors associated with early sexual intercourse which included general characteristics of participants, substance use, information surrounding to their early sexual intercourse

: We have made more alignment in the whole text. Thank you very much for the great comment.

5. The Methods section need to be reorganized. There are too many one-sentence sub-headed sections.

: Thank you for the great comment. We have re-organized the whole section of the method.

6. Why was it only necessary to "detect the reliability of the last part of the questionnaire" and not the other sections

: Because the questions in part no1-3 are the questions asking such as age, sex, and education. In the last past, questions were asked about the knowledge and attitude, which are required to detect reliability. Reliability concerns the extent to which a measurement of phenomena provide stable and consistent result or repeatable of the tool.

: However, the whole set of the questionnaire was detected for its validity by piloted and expert review or IOC methods. The reliability test is tested for how well the tool is able to collect the actual information or existing information from the participants.

Reference: Taherdoost H. Validity and reliability of the research instrument: how to test the validity and reliability of a questionnaire/survey in research. International Journal of Academic Research in Management. 2016; 5(3): 28-36.

7.Please clarify and justify if indeed the sampling basically involved a census of all eligible youth in each of the 30 selected villages?

: Thank you very much, we have added information in the process of study population selection on page 4, lines 4-6, and 11-13.
8.Why is "Illiterate" considered a category for the variable on education. The measure of literacy differs from that of educational level/attainment or school attendance.

: Sorry for the misuse of the word. We refer to “no education” or “never attended a school”. Thank you for the great comment.

9.Please define "one-night stand"

: One-night stand is the action of having sex without any other relationship, and also they have a little period of time to know each other prior to following references;

No.1 Dolan AM, Rosenberg L, starin JL, Welsh M. One nightstand. Available from: <http://danieljglendening.com/files/one-nightstand.pdf>.

No. 2 Christianson M, Johansson E, Ekkelin M, Westman G. “One-night stands” -risky trops between lust ans trust: qualitative interviews with Chlamydia trachomatis youth in north Sweden. Scand J Public Health. 2003; 31: 44-50.

10. I do not think Table 2 presents any univariate analyses as the heading seems to suggest

: All the OR, 95%CI, and p-value in columns no.4-6 were calculated by the simple logistic regression or univariable analysis.

: Sorry if I used some mistake forms of terminology.

11. Please clarify what you mean by Level of knowledge and Level of attitude and provide details of what constituents formed their assessments and rating/ranking.

: Thank you the levels of knowledge and attitude have been explained in the section of “variable”, between page 5, lines 27-34, and page 6, lines 1-2.

12. The discussion needs to be thoroughly improved.

: Thank you for the comment. We have made almost the whole part of the discussion part. We very much hope that you are happy with this version.

Reviewer #2: Reviewer Comments to Authors
The study sought to explore the sexual behaviours and associated factors to early sexual initiation among the youths of hill tribe in Thailand. It is a very good study whose findings have implications for achieving improved reproductive and sexual health for the youth across the region.
1. Abstract
The abstract provides a sufficient summary to the study with the objective of the study clearly stated.

: Thank you so much.

2. Introduction
The background is well-written and the research question clearly and completely defined. This has also pointed out the fact that the research question has not been resolved. Problem is clearly stated and aligns with the background provided. However, the authors should define what they mean by “unintended sexual intercourse”.

: Thank you for the great comment on this point. After carefully checking the context and the context including the reference, this is a mistake while writing up the manuscript. We are so sorry!

3. Methods
The methods section provides adequate description and information for repeating the study. Nevertheless, the following issues should be address:
• Authors should provide justification for the study design used

: We used a cross-sectional design (page 3, lines 24-27) because we need to assess both outcomes (early sexual intercourse) and several risk factors at the same time. A cross-sectional design is good for exploring a relationship in any context that no specific information prior. The outcomes could be looking for several associations from variables.

: The cross-sectional is known as a rapid method and less expensive compared to cohort study and also given the prevalence of the outcome among the population.

: Within the context of sexual behaviors among the hill tribe youths, it would be one of the fittest methods to determine the factors associated with the outcome (early sexual intercourse).

• Authors should state the sample size formula in the work for easy reference using the stated assumed values of p, q, and e.

: Thank you very much for such a wonderful comment. The formula was drawn with definitions in the paper on page 4, lines 10-13.

4. Results
The results have been clearly presented. However, no reference to a table or figure is made for the results narrative after table 1. It is therefore, difficult for one to follow the narrative without the authors indicating where (figure or table or supporting file) the narratives are based on. This should be addressed and reconciled.

: Sorry, we have added new tables (tables 2 and 3) to present the information.

: The context on pages 6-8, and their summaries.

5. Discussion
The Discussion is succinct and the authors have provided explanations or proffered possible reasons to explain their observations made.

: Thank you.

6. Conclusion
The conclusions and recommendations reflect the study’s findings. However, the authors failed to indicate the implications of the key findings made which support the recommendations made. Authors should revise as such.

: Sorry, we have revised the whole section of the conclusion to reflect the findings with appropriate recommendations.

6. PLOS authors have the option to publish the peer review history of their article ([what does this mean?](https://journals.plos.org/plosone/s/editorial-and-peer-review-process#loc-peer-review-history)). If published, this will include your full peer review and any attached files.


If you choose “no”, your identity will remain anonymous but your review may still be made public.


**Do you want your identity to be public for this peer review?** For information about this choice, including consent withdrawal, please see our [Privacy Policy](https://www.plos.org/privacy-policy).

Reviewer #1: No

Reviewer #2: No

Regards,

TK

Assist Prof. Dr.Tawatchai Apidechkul, Dr.P.H.(Epidemiology)

Former Hubert H Humphrey Fellow (Fulbright)

Director The Center of Excellence for the Hill tribe Health

Associate Dean, School of Health Science,

Mae Fah Luang University, Thailand

Tel.+66-53916914

EmaiL:Tawatchai.api@mfu.ac.th
