# Supplementary material for: Factors influencing early sexual initiation among hill tribe youths in Chiang Rai Province, Northern Thailand: A community-based cross-sectional study
Source: PLoS One. 2025 Apr 8;20(4):e0321083. doi: 10.1371/journal.pone.0321083 (PMC11991288; doi:10.1371/journal.pone.0321083)
Supplement: Attachment — Submitted filename: PLOS ONE_early sexual initiation.docx [file pone.0321083.s004.docx]

PONE-D-23-33898

Influencing factors early sexual initiation among hill tribe youths in northern Thailand

PLOS ONE

Dear Editor,

Thank you very much for opportunity to review the manuscript titled " Influencing factors early sexual initiation among hill tribe youths in northern Thailand" for PLOS ONE.

I think that some aspects of this manuscript are interesting. I believe that some major changes are required. Below are my comments about this manuscript, which I hope are some assistances to the authors.

**1.The title**, author should be specific “in Chiang Rai province”? not representative in northern of Thailand, and may considering add approach “: community based cross sectional approach”

**2.An abstract**

- Author conclusions “A large proportion of the hill tribe youths have experienced early sexual initiation before the age of 15 years. A specific training program should be integrated into the high school study curriculum to protect early sexual initiation behaviors and safe sex, particularly in males. The curriculum should focus on the impact of alcohol and working in unskilled work sectors and potentially early sexual initiation.

As the result based author may conclude in community or family based intervention, attitude come from socialization in family….etc. Thailand has a sexual education curriculum at all level in school.

**2. Methodology,**

- page 5 line 25-27 author mention that “In the section on knowledge of safe sex (10 items), there were classified into three categories; poor (0-5 scored), moderate (6-8 scored), and good (9-10 scored).” It is knowledge about HIV transmission (not safe sex) Please clearly how scoring and range of score and cutting point? Including reference?

-Substance use question between Thai and English inconsistent. The answer means? Current use? Ever but quit? Never or ever tried? Please clearly explain at variable section. What yes?

**3. Results**

- Table 3 Something error? Author present variable” Alcohol or drug use prior having sexual initiation with 3 answers <no, sometime, yes> but in questionnaire part 3 item 3.9 in English and Thai language the answer only <Yes, No>

- Table 1 and 2 author should present as below

| **characteristics** | **Total** | | **Early sexual initiation** | | | | **Never had sexual experience** | | **χ 2** | **p-value** |
| --- | --- | --- | --- | --- | --- | --- | --- | --- | --- | --- |
|  |  |  | **Yes (<14)** | | **No (**≥**15)** | |  |  |  |  |
|  | **n** | **%** | **n** | **%** | **n** | **%** | **n** | **%** |  |  |
|  |  |  |  |  |  |  |  |  |  |  |
|  |  |  |  |  |  |  |  |  |  |  |

-Table 4 Author definition “Early sexual initiation” yes= age at first sexual initiation <14 and No= age at first sexual initiation ≥15 including never had sexual experience. Author should exclude never had sexual experience or use as reference.

**4. Discussion**

Page 15-line 16-18 Author discuss that “The hill tribe youths have a younger age of their first sexual initiation than Thai youths which might be the culture of the hill tribe that needs to get married early to have a child for help in their family work.”

Is it culture issue? Economic or occupational Issue? Author should explain how culture difference from hill tribe and general youths.
